# Integrating longitudinal clinical laboratory tests with targeted proteomic and transcriptomic analyses reveal the landscape of host responses in COVID-19

**DOI:** 10.1038/s41421-021-00274-1

**Published:** 2021-06-08

**Authors:** Yun Tan, Wei Zhang, Zhaoqin Zhu, Niu Qiao, Yun Ling, Mingquan Guo, Tong Yin, Hai Fang, Xiaoguang Xu, Gang Lu, Peipei Zhang, Shuangshuang Yang, Ziyu Fu, Dongguo Liang, Yinyin Xie, Ruihong Zhang, Lu Jiang, Shuting Yu, Jing Lu, Fangying Jiang, Jian Chen, Chenlu Xiao, Shengyue Wang, Shuo Chen, Xiu-Wu Bian, Hongzhou Lu, Feng Liu, Saijuan Chen

**Affiliations:** 1 Shanghai Institute of Hematology, State Key Laboratory of Medical Genomics, National Research Center for Translational Medicine at Shanghai, Ruijin Hospital Affiliated to Shanghai Jiao Tong University School of Medicine, Shanghai, China; 2 School of Life Sciences and Biotechnology, Shanghai Jiao Tong University, Shanghai, China; 3 Shanghai Public Health Clinical Center, Fudan University, Shanghai, China; 4 Intelligent Pathology Institute, Division of Life Sciences and Medicine, University of Science and Technology of China (USTC), and Department of Pathology, The First Affritted Hospital of USTC, Hefei, Anhui China; 5 Department of Pathology, Shanghai Jiao Tong University School of Medicine, Shanghai, China; 6 Department of Thoracic Surgery, Shanghai Pulmonary Hospital, Tongji University School of Medicine, Shanghai, China; 7 Department of Laboratory Medicine, Ruijin Hospital, Shanghai Jiao Tong University School of Medicine, Shanghai, China; 8 Institute of Pathology and Southwest Cancer Center, Southwest Hospital, Third Military Medical University (Army Medical University), Key Laboratory of the Ministry of Education, Chongqing, China

**Keywords:** Immunology, Cell signalling, Proteomics

## Abstract

The pathophysiology of coronavirus disease 19 (COVID-19) involves a multitude of host responses, yet how they unfold during the course of disease progression remains unclear. Here, through integrative analysis of clinical laboratory tests, targeted proteomes, and transcriptomes of 963 patients in Shanghai, we delineate the dynamics of multiple circulatory factors within the first 30 days post-illness onset and during convalescence. We show that hypercortisolemia represents one of the probable causes of acute lymphocytopenia at the onset of severe/critical conditions. Comparison of the transcriptomes of the bronchoalveolar microenvironment and peripheral blood indicates alveolar macrophages, alveolar epithelial cells, and monocytes in lungs as the potential main sources of elevated cytokines mediating systemic immune responses and organ damages. In addition, the transcriptomes of patient blood cells are characterized by distinct gene regulatory networks and alternative splicing events. Our study provides a panorama of the host responses in COVID-19, which may serve as the basis for developing further diagnostics and therapy.

## Introduction

Since its appearance in December 2019, COVID-19 has become arguably the most challenging pandemic since the 1918 flu^1^. The causative pathogen, severe acute respiratory syndrome coronavirus 2 (SARS-CoV-2), is highly infectious and uses its spike (S) protein to bind with the host ACE2 receptor for cell entry^2,3^. As of 11th April 2021, SARS-CoV-2 has infected over 134 million people and a large body of evidence indicates that the virus has been undergoing constant evolution (https://covid19.who.int/). In more than 1060,000 sequenced viral genomes, a number of viral clades could be distinguished, and a dominant D614G mutation in the S gene (https://www.gisaid.org/), often accompanied by other mutations, such as B.1.1.7, B.1.351, and B.1.1.28.1, has emerged that either increases the viral infectivity or escapes the immunity against the prototype of the virus^4–6^. Nevertheless, the impact of these sequence changes on the severity of COVID-19 remains to be investigated. So far, it seems the main known determinants of the disease phenotypes still reside in host factors such as age, sex, comorbidity, blood groups, and the integrity of immune system^7–11^.

Like many types of respiratory infections, including the flu, COVID-19 exhibits a spectrum of symptoms in patients. In terms of pathophysiology, several stages of disease progression can be identified, from the initial upper respiratory tract infection when the affected individuals are asymptomatic but capable of viral transmission, to the infection of alveolar epithelial cells in the lung and ensuing pneumonia, and the eventual damages of the pulmonary epithelial–endothelial barrier due to hyperinflammation^3,12–16^. While most COVID-19 patients either remain asymptomatic or only develop upper respiratory tract symptoms, in ~5%–10% cases, the disease deteriorates, leading to systemic disturbance of the immune system and multiorgan failure^9,17^. Studies using cell culture, animal models, and patient samples have shown that SARS-CoV-2 generally induces weak antiviral responses and elevated expression of chemokines and cytokines including IL-6^18,19^, and it is only in patients with severe/critical symptoms that lymphocytopenia and cytokine release syndromes (CRS) become evident^20–23^. Given its role in heightened inflammation, acute respiratory distress syndrome (ARDS) and vital organ damages, CRS has been implicated as a main cause of severe/critical COVID-19. However, the molecular and cellular mechanisms underlying the initiation of CRS needs further exploration, and the relationship between COVID-19 severity and host response determinants in the majority of cases remain unclear.

The human immune system is endowed with multiple immune cell types and a cascade of cellular and molecular interactions to counter against invading pathogens, including newly emerged coronaviruses. Thus, a deep understanding of the pathophysiology of COVID-19 necessitates detailed spatial and temporal examination of the factors during SARS-CoV-2 infection and recovery^24–26^. Following this line of investigation, we have systematically analyzed clinical laboratory parameters in blood samples collected from 963 COVID-19 patients during their hospital stay (median: 14 days (IQR 10–20)). We also performed a suite of molecular and cellular studies by means of targeted proteomics and steroid hormone assays in a part of these samples, and delineated the relative order of the changes of circulatory factors and immune cell composition during the course of disease progression. Furthermore, we compared the transcriptomes of patient-derived bronchoalveolar lavage fluid (BALF) and peripheral blood mononucleated cells (PBMCs, containing mainly lymphocytes and monocytes) samples to trace the origin of aberrant circulating cytokines, chemokines, and other secreted proteins. Finally, we analyzed the activities of gene regulatory networks and the usage of alternative splicing (AS) in the PBMC during the infectious phase of COVID-19. Together, our study provides a comprehensive and dynamic view of the host response during the development of COVID-19.

## Results

### Comprehensive profiling of the dynamics of host responses of COVID-19 using longitudinal clinical laboratory tests

**Fig. 1:**
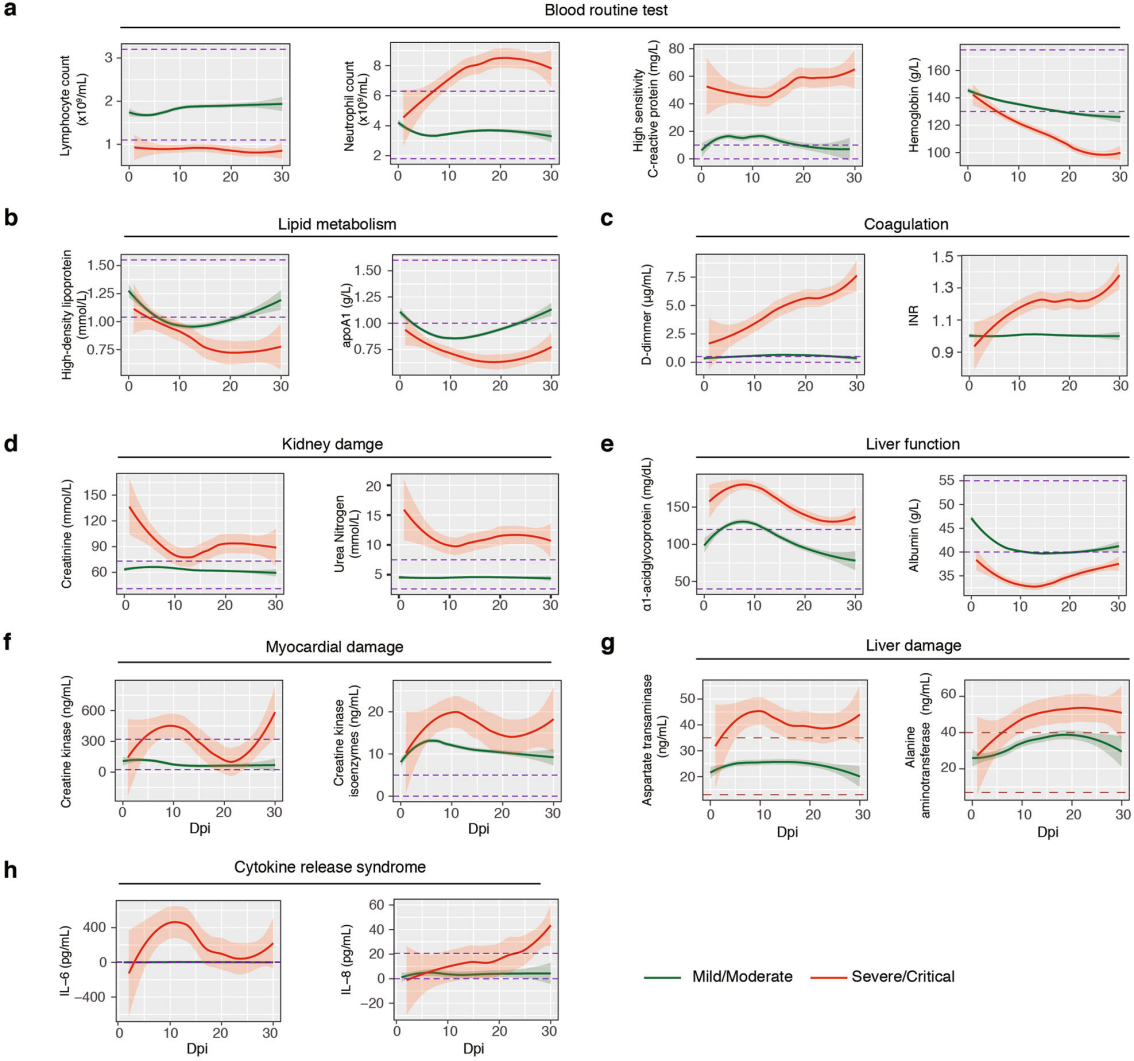
The dynamics of host responses in COVID-19. **a** Longitudinal analysis of a subset of routine blood test results from 1 to 30 dpi in mild/moderate and severe/critical cases of COVID-19, including lymphocyte count, neutrophil count, high sensitivity C-reactive protein level, and hemoglobin level. The numbers of cases and samples per test are shown below each panel. Green represents the mild/moderate group; red represents the severe/critical group. Details of statistical analyses are shown in Supplementary Table S2. A complete summary of routine blood test results are shown in Supplementary Fig. S1a, b. A total of 925 mild/moderate cases (3516 samples) and 32 severe/critical cases (548 samples) were used for plotting. **b** Longitudinal analysis of lipid metabolism in COVID-19 patient serum. The levels of high-density lipoprotein and apoA1 lipoprotein are shown. Additional measures are shown Supplementary Fig. S1c. A total of 563 mild/moderate cases (1818 samples) and 26 severe/critical cases (120 samples) were used for plotting. **c** Longitudinal analysis of coagulation-related features in COVID-19 patient serum. The levels of D-dimer and International normalized ratio (INR) are shown. Additional measures are shown in Supplementary Fig. S3. A total of 921 mild/moderate cases (3067 samples) and 32 severe/critical cases (483 samples) were used for plotting. **d–g** Longitudinal analysis of kidney damage, liver function, myocardial damage, and liver function related features in COVID-19 patient serum. The levels of creatinine, blood urea nitrogen, albumin, α-acid glycoprotein, creatine kinase, and creatine kinase isoenzymes, aspartate aminotransferase, and alanine aminotransferase are shown. Additional measures are shown in Supplementary Fig. S3. A total of 924 mild/moderate cases (3445 samples) and 32 severe/critical cases (495 samples) were used for plotting. **h** Longitudinal analysis of cytokine release syndrome (CRS)-related features in COVID-19 patient serum. The levels of IL-6 and IL-8 are shown. A total of 818 mild/moderate cases (1675 samples) and 30 severe/critical cases (177 samples) were used for plotting.

To systematically track the abnormality of host responses at different stages of COVID-19, we analyzed a number of biomarkers associated with systemic responses to SARS-CoV-2 in the peripheral blood samples of 963 patients (including 33 severe/critical and 930 mild/moderate cases) during both infection and convalescence stages (see Supplementary Table S1 for main patient demographic and clinical characteristics). First, we performed a time-course analysis of routine blood tests in different severity groups (mild/moderate versus severe/critical) within the first month after appearance of symptoms. Compared to mild/moderate cases, the lymphocyte counts and blood lymphocyte percentages in severe/critical COVID-19 were consistently lower from 1 to 30 days post-illness onset (dpi) (Fig. 1a; Supplementary Table S2). The levels of hemoglobin (Hb) and hematocrit (HCT) were initially the same in all patient samples, but then progressively reduced in both groups, with severe/critical cases showing more profound decreases (Fig. 1a; Supplementary Table S2). By contrast, the neutrophil counts and blood neutrophil percentages appeared normal at the early stage of severe/critical COVID-19, and then gradually increased between 10 and 28 dpi (Fig. 1a; Supplementary Table S2). These results indicated that neutrophil activation and anemia slightly lag behind acute lymphocyte losses. After about 1 month post-illness onset, when severe/critical patients were in convalescence, the blood counts of lymphocytes and neutrophils gradually regressed to normal ranges, but those of Hb and HCT, as well as the levels of lipid proteins, including high-density lipoprotein (HDL) and apoA1 (Fig. 1b; Supplementary Fig. S1 and Table S2), remained low for up to 3 months (Supplementary Fig. S1).

Shortly after the appearance of illness, D-Dimer (D-D), activated partial thromboplastin time (APTT), fibrin/fibrinogen degradation products (FDP), and the international normalized ratio (INR) showed gradual increase in cases that deteriorated into severe/critical conditions while remaining low in mild/moderate cases (Fig. 1c; Supplementary Fig. S2 and Table S2). These observations were in consistence with our finding in the autopsies of a separate series of deceased COVID-19 patients (Supplementary Fig. S3) and previous reports^27–30^, where intensive thrombi in the pulmonary, hepatic, nephrotic, and myocadiac tissues were noticed. Coagulation markers gradually restored to relatively normal ranges after 2 months of infection in severe cases (Supplementary Fig. S3).

For markers associated with kidney injury, the levels of creatinine (Cr), blood urea nitrogen, and cystatin C were abnormally high in severe/critical COVID-19 as early as 1–2 dpi, followed by a rapid decrease before becoming plateaued after 10 dpi (Fig. 1d; Supplementary Table S2). For markers of liver function, the levels of alkaline phosphatase (ALP), prealbumin, and albumin (ALB) were significantly lower in severe/critical COVID-19 at 1–2 dpi as compared to mild/moderate COVID-19 (Fig. 1e; Supplementary Table S2). For markers correlated with liver and myocardial damages, the levels of aspartate aminotransferase (AST), alanine aminotransferase (ALT), creatine kinase (CK), and creatine kinase isoenzyme (CK-MB) were relatively normal at the onset of illness; later, they temporarily rose and decreased between 3 and 21 dpi, before rising again at 21–30 dpi (Fig. 1f, g; Supplementary Table S2). The levels of these organ injury makers returned to normal ranges after 2 months except for that of Cr, which entered a second phase of increase until as late as 90 dpi (Supplementary Fig. S3).

Previously, elevated cytokines such as IL-6 have been identified as hallmarks of severe/critical cases of COVID-19^31^. Examination of IL-6, IL-8, and some other cytokines over time revealed highly dynamic changes in patient serum, which fell into two broad groups. The first was characterized by IL-6, whose level was relatively normal at the onset of illness, and then increased and peaked at about 10 dpi (Fig. 1h; Supplementary Table S2). The other group was characterized by IL-8, whose level gradually increased between 1 and 30 dpi in severe/critical cases while remaining low in mild/moderate cases (Fig. 1h; Supplementary Table S2). During convalescence, the levels of these cytokines were within normal ranges (Supplementary Table S2).

Together, these results delineated the relative order of key host responses deviated in COVID-19 patients. In particular, compared to healthy controls and mild/moderate cases, the progression of severe/critical COVID-19 starts with acute lymphocytopenia and signs of kidney injuries, followed by seemingly dynamic production of cytokines and increased levels of neutrophils, coagulation, and signs of heart failure. Most of these host responses subsided over time, except that anemia and coagulation abnormalities persisted in some cases who recovered from severe/critical conditions (Fig. 1b; Supplementary Figs. S1, S3 and Table S2), which might be associated with residual symptoms during post-SARS-CoV-2 convalescence. In agreement with previous reports^9,32,33^, age and systemic immune responses emerged as independent risk factors (Supplementary Table S3). In addition, six patients from the critical/severe group died during the course of this study. The abnormalities of most routine blood parameters and immune cell counts and ratios were generally more pronounced in these deceased cases than in survived severe/critical ones. Furthermore, for the deceased cases, the Cr, urea nitrogen, and cystatin C levels were dramatically increased at the early stages post-SARS-COV-2 infection, whereas the CK, CK-MB, and IL-6 levels were much more strongly increased at 8–15 dpi (Supplementary Fig. S4).

### Longitudinal profiling of cytokines and chemokines in patient serum

**Fig. 2:**
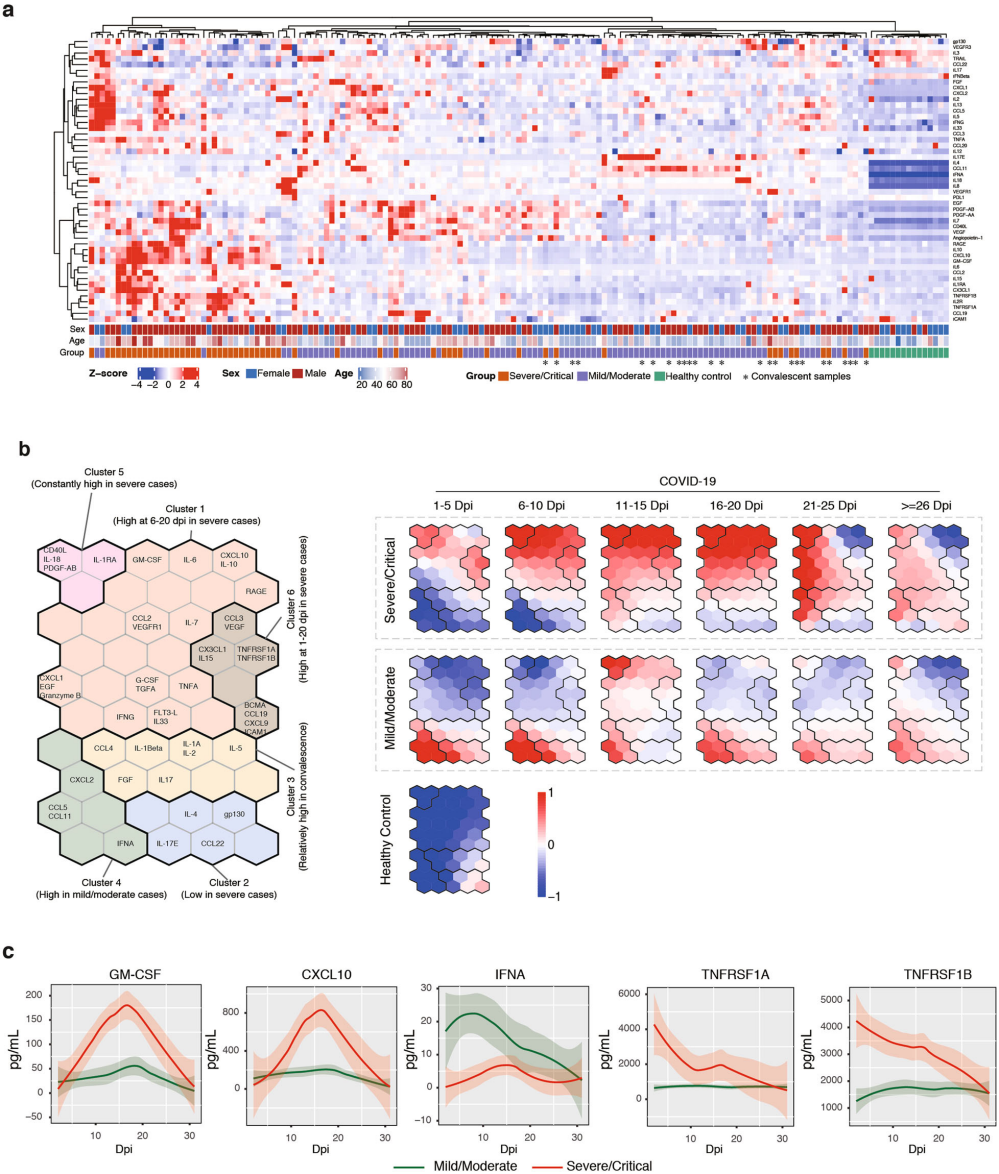
Comprehensive profiling of cytokines and chemokines in COVID-19. **a** Heatmap shows the relative levels of 62 cytokine and chemokines in COVID-19 serum samples. The age, stage (I: 1–10 dpi; II: 10–30 dpi, III: > 30 dpi), and the severity of each case were shown at the bottom. The matrix of raw data is shown in Supplementary Table S3. **b** Self-organizing map of five clusters of cytokines and chemokines in COVID-19 samples. The samples were divided into six time-coursed (1–5, 6–10, 11–15, 16–20, 21–25, > 26 dpi). The relative levels of cytokines/chemokines were plotted for each patient group (severe/critical and mild/moderate) on the right. Healthy samples were used as controls. Red represents higher levels and blue represents lower levels. **c** Dynamic changes of the serum levels of GM-CSF, CXCL10, IFNA, TNFSF1A, and TNFSF1B within the first 30 dpi. A total of 51 mild/moderate cases (90 samples) and 24 severe/critical cases (56 samples) were used for plotting.

To systematically investigate potential factors contributing to the dynamic host responses in COVID-19, we applied the Luminex cytokine profiling assay to analyze a panel of 62 cytokines in the serum of 146 COVID-19 samples (mild/moderate, 90 samples/51 patients; severe/critical, 56 samples/24 patients) and 15 healthy donors (Fig. 2a; Supplementary Fig. S5 and Table S4). In consistence with previous observations^9,34,35^, IL-6, CCL2, CXCL2, CXCL10, and IL-8 were significantly elevated in COVID-19 samples (Supplementary Fig. 5a). Compared to mild/moderate cases, severe/critical samples also contained significantly higher levels of TNFRSF1A, TNFRSF1B, ICAM-1, IL-6, CXCL10, GM-CSF, CX3CL1, IL-2R, IL-1RA, and VEGF-R1 (Supplementary Fig. S5b). Moreover, the levels of TNFRSF1A, TNFRSF1B, IL-1RA, IL-2R, VEGF-R3, PDGF-AA, IL-8, and IL-18 remained high in convalescent samples than in the healthy controls (Supplementary Fig. S5c). Thus, the profiles of these cytokines correlated with the severity and stage of COVID-19 during both infection and recovery.

To more closely examine the temporal changes of cytokines and chemokines, we compared their levels at six time windows during the first 30 dpi. A self-organizing method indicated that, between mild/moderate and severe/critical cases, many cytokines and chemokines showed significant differences at the early stages of illness onset. For example, on 1–10 dpi, the levels of IFN-α, IL-17E, CCL5, CXCL2, and CCL11 were high in mild/moderate cases but not in severe/critical ones (Fig. 2b, Clusters 2 and 4). IFN-α is a known regulator of antiviral responses^36^, and IL-17E (also known as IL-25) regulates T helper cell functions via activating type 2 T helper cells^37,38^. The low production of them might thus contribute to a weakened cellular response in severe COVID-19. An opposite trend was observed for TNFRSF1A, TNFRSF1B, CD40L, IL-18, PDGF-AB, and IL-1RA, whose levels were high in severe/critical cases but not in mild/moderate ones between 1 and 10 dpi (Fig. 2b, Clusters 1, 5, and 6). The serum TNFRSF1A and TNFRSF1B, or solTNFR1 and solTNFR2, are respectively derived from ectodomain shedding of transmembrane receptors of TNF-α (e.g., TNFRSF1A and TNFRSF1B)^39^. Previous studies showed that TNF/TNFR2 interaction could suppress T-cell response in the lung during acute influenza infection^40,41^. Therefore, the overproduction of these cytokines and chemokines might be indicative of impaired T-cell response in the alveoli in severe/critical COVID-19.

Furthermore, most CRS-related cytokines, including IL-6, GM-CSF, CXCL10, IL-10, receptor for advanced glycation end (RAGE products), CCL2, VEGRF1, IL-7, CCL3, VEGF, CX3CL1, and IL-15, were highly produced between 6 and 20 dpi in severe/critical cases (Fig. 2b, c), suggesting that CRS mainly occurred within this time window. After 26 dpi, the majority of cytokines and chemokines returned to baseline levels, and there was little difference between different severity groups at convalescence (Fig. 2b; Supplementary Fig. S5), suggesting a gradual recovery from abnormal cytokine release-associated symptoms.

### Mechanisms underlying lymphocytopenia in severe/critical COVID-19

**Fig. 3:**
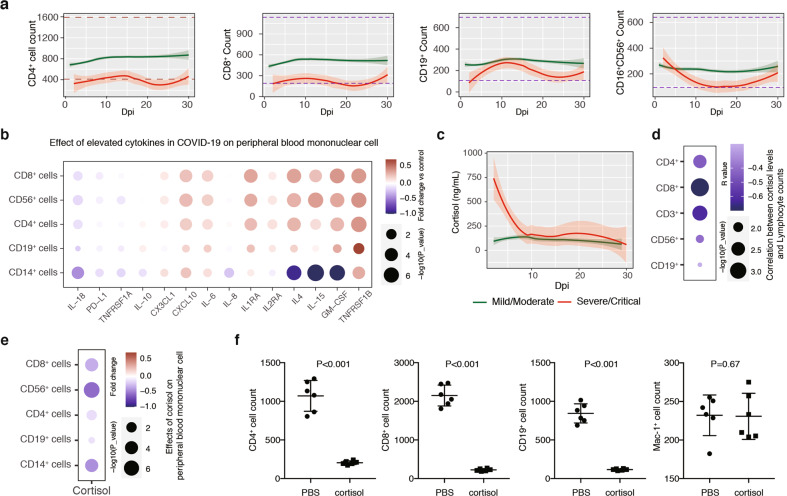
The role of cytokines and cortisol in acute lymphocytopenia in severe/critical COVID-19. **a** Longitudinal analysis of major subsets of lymphocytes in COVID-19 patients. The counts of CD4^+^, CD8^+^, CD19^+^, and CD16^+^CD56^+^ cells were shown. Pink indicates severe/critical cases; green indicates mild/moderate cases. **b** Summary of PBMC-treatment assay. PBMCs from healthy donors were treated with indicated cytokines and circulatory factors for 36 h. The relative cell counts of CD4^+^, CD8^+^, CD19^+^, CD14^+^, and CD56^+^ cells were shown. **c** Longitudinal analysis of serum cortisol levels in COVID-19 patients. A total of 24 mild/moderate cases (38 samples) and 16 severe/critical cases (38 samples) were used for plotting. **d** Correlation between cortisol levels and the counts of CD3^+^, CD4^+^, CD8^+^, CD19^+^, and CD56^+^ cells. Color represents Pearson correlation coefficient values and circle sizes represent −Log10 (*P* values). **e** Effects of cortisol on CD4^+^, CD8^+^, CD19^+^, and CD56^+^ cells in PBMC-treatment assay. **f** CD4^+^, CD8^+^, CD19^+^, or Mac1^+^ cell counts in mice treated with PBS or cortisol.

Acute lymphocytopenia is by far the most reliable prognostic feature of severe/critical COVID-19^20,42^. By monitoring distinct subsets of lymphocytes over time, we found that lymphocytopenia occurred early and persisted in severe/critical COVID-19 (Fig. 3a; Supplementary Fig. S1 and Table S2), and there was a slight increase of T-cell counts (including CD4^+^ and CD8^+^ T cells) in severe/critical cases at 10–15 dpi, followed by a decrease between 15 and 22 dpi and a second minor increase thereafter. A similar pattern of fluctuation was observed for the B cells (CD19^+^) (Fig. 3a). Of note, lymphocytopenia in severe COVID-19 was suggested to be mechanistically linked with CRS^43^. However, the temporal profiles of major lymphocyte subsets and CRS-related cytokines did not show a simple association, since the onset of cytokine production lagged behind acute lymphocyte losses by ~6 days, and they both showed transient increases between 6 and 20 dpi (compare Figs. 1h and 3a).

To functionally test the role of cytokines on the viability of lymphocytes, we treated PBMCs isolated from healthy donors with selected groups of cytokines (e.g., IL-10, IL-15, IL-18, IL-8, IL-6, IL-1RA, IL-2RA, IL-4) for 36 h, using the maximum doses detected in COVID-19 serum. In addition, a group of elevated chemokines and inflammatory factors was included in this assay (e.g., CX3CL1, CXCL10, GM-CSF, PD-L1, TNFRSF1A, and TNFRSF1B). We found that the majority of them tended to increase the number of lymphocytes (CD4^+^, CD8^+^, or CD56^+^), including CX3CL1, CXCL10, GM-CSF, IL-15, IL-6, IL-1RA, IL-4, and TNFRSF1B (Fig. 3b); only IL-18 treatment led to weak reduction of CD4^+^, CD8^+^, and CD56^+^ cells. These results thus did not support a critical role for individual cytokines and chemokines in causing acute lymphocytopenia at the onset of illness in severe/critical COVID-19, although we could not exclude the possibility that these factors may be able to affect lymphocytopenia in the presence or absence of additional factors in vivo.

We also examined the serum levels of two endogenous steroid hormones, cortisol and aldosterone, in patient samples over time. We found that the level of cortisol, but not aldosterone, was abnormally high in the first 1–3 dpi specifically in severe/critical cases, which then rapidly decreased to baseline level as in the mild/moderate cases (Fig. 3c; Supplementary Fig. S6). In addition, the levels of cortisol were negatively correlated to the lymphocyte counts (Fig. 3d), while no exogenous steroid hormone drug had been used in this period. When PBMCs from healthy donors were treated in vitro with cortisol at the averaged maximal levels in severe/critical COVID-19, the numbers of CD8^+^, CD56^+^, and CD14^+^ cells were significantly reduced (Fig. 3e). Severe reduction of lymphocytes was also observed within 36 h in the peripheral blood of mice, when the latter were treated with cortisol at a dose corresponding to the level in severe/critical COVID-19 patients (Fig. 3f), which is in support of a role for elevated cortisol in causing lymphocytopenia^44–46^.

### Networks of serum proteins correlated with disease severity and multiorgan damages

To systematically investigate the relationship between circulating factors and systemic organ injuries in COVID-19, we used a targeted proteomics approach, namely the O-link proximity extension assay, to measure 304 secreted proteins engaged in immune response, inflammation, cardiovascular regulation, and organ damage in 77 COVID-19 serum samples (34 samples from 17 mild/moderate cases; 43 samples from 21 severe/critical cases), and 8 healthy control samples (Supplementary Table S5). Many immune response factors in the O-link assay were also included in the above Luminex-based cytokine/chemokine profiling experiments, and the results using both methods were highly accordant with each other (Supplementary Fig. S7).

**Fig. 4:**
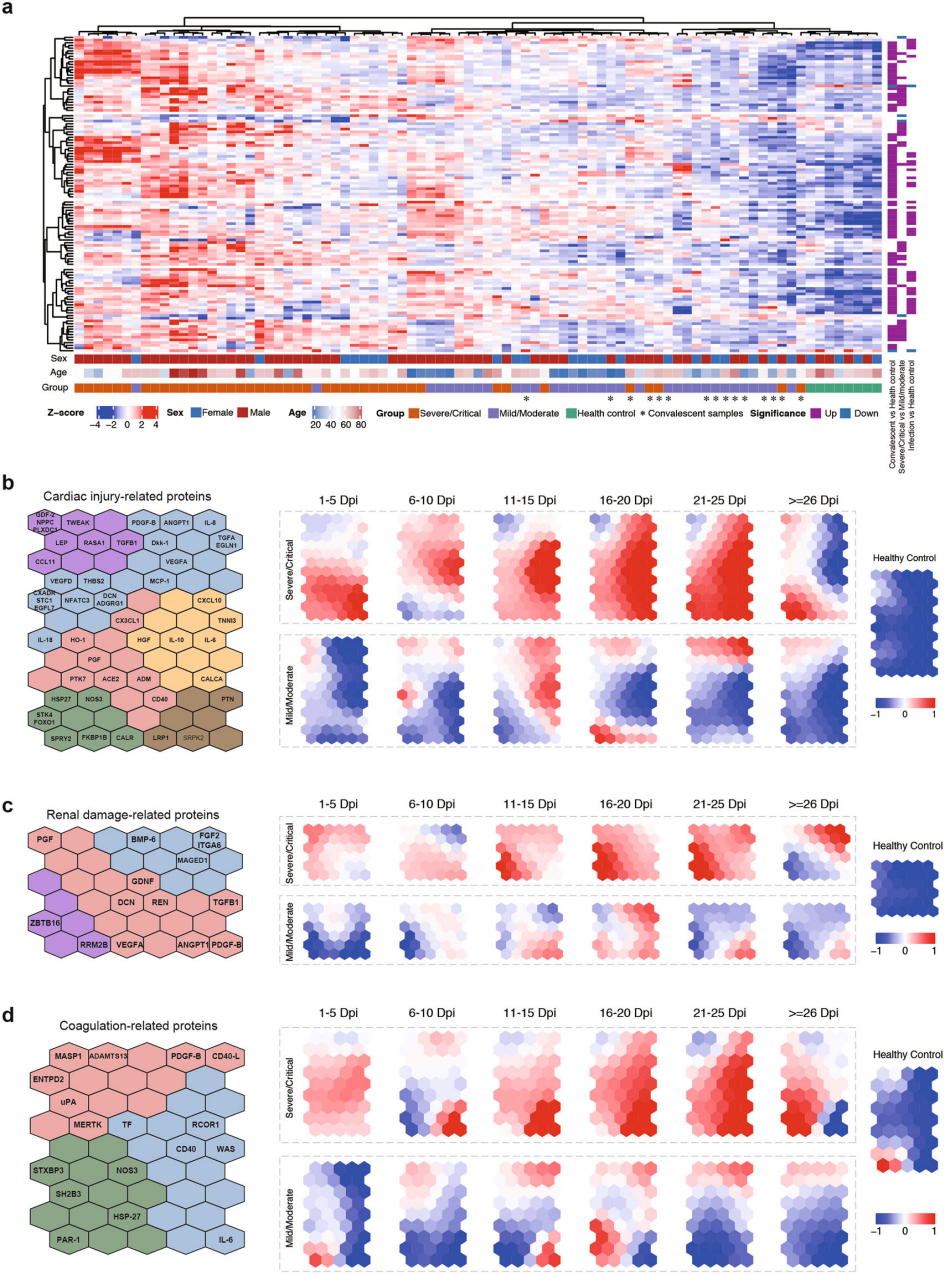
Targeted proteomics of serum proteins in COVID-19. **a** Heatmap of the relative levels of serum proteins related to immune response, organ damage, cardiovascular disease, and inflammation response in ten healthy donors and 88 COVID-19 cases. The matrix of raw levels was shown in Supplementary Table S3. **b**–**d** Dynamical changes of proteins related to cardiovascular disorder (**b**), renal and hepatocellular damage (**c**), and coagulation (**d**) in COVID-19 cases. Self-organizing maps of factors in each group were shown on the left. The relative levels of each protein over time in different patient groups (severe/critical and mild/moderate) were shown on the right. The samples were divided into six time windows (1–5, 6–10, 11–15, 16–20, 21–25, > 26 dpi). Healthy donor samples were used as controls. Red represents higher levels and blue represents lower levels.

Unsupervised clustering analysis indicated widespread increase of circulatory proteins in COVID-19 as compared to healthy controls (Fig. 4a; Supplementary Fig. S8 and Table S5). The majority of them were also expressed at higher levels in severe/critical cases than in mild/moderate ones, including those related to cardiovascular abnormalities (e.g., CEACAM8, ANGPT1, soluble CD40L, and DKK-1), immune responses and inflammation (e.g., DCTN1, GLB1, PRDX5, and ST1A1), organ damage (e.g., FKBP1B, PTPRJ, MAEA, and BTC), and coagulation (e.g., tissue factor (TF)).

We divided patient samples into six time windows and used the self-organizing mapping method to analyze the dynamic changes of proteins involved in different types of organ damages (e.g., cardiovascular and renal damages) and coagulation (Fig. 4b–d). In each case, distinct groups of proteins were increased in waves within the first 30 dpi. The time between 6 and 25 dpi appeared as a critical window, whereby most markers of organ damage and coagulation showed a steady and coordinated rise, including TNNI3 and HGF for cardiac injury (Fig. 4b), ZBTB16 and RRM2B/P53R2 for renal damage (Fig. 4c), and TF, RCOR1, WAS, and IL-6 for coagulation (Fig. 4d)^47,48^. These observations were largely in agreement with the aforementioned laboratory tests, which showed consistent widening of the levels of many biomarkers in the same time frame (Fig. 1b–g). In addition, this analysis revealed a number of regulators previously reported in various pathophysiological processes. For example, PTN was shown to potentiate the apoptosis of cardiomyocyte^49^ and its early elevation (1–5 dpi) in severe COVID-19 might be a cause for subsequent cardiac injury (Fig. 4b). FGF2, also known as basic fibroblast growth factor, was found to induce strong apoptosis of kidney cells during MERS-CoV infection^50^. In severe COVID-19, FGF2 level showed small fluctuations between 1 and 20 dpi, and then rapidly increased after 21 dpi, which might be responsible for SARS-CoV-2-triggered renal damage as well (Fig. 4c). In a similar manner, the TF showed a transient increase within 5 dpi, and then increased to high levels between 11 and 25 dpi (Fig. 4d). Given its role in initiating extrinsic coagulation and thrombin formation, high level TF may be responsible for the observed intensive microthrombi in deceased severe COVID-19 cases (Supplementary Fig. S3). Together, these results identified a large number of circulatory proteins whose dynamic changes in COVID-19 serum might contribute to or reflect the course of multiorgan damage, aberrant coagulation, and some other pathophysiological processes.

### Disturbed antiviral immune response and aberrant cytokines/chemokines production in the alveolar microenvironment of severe/critical COVID-19

**Fig. 5:**
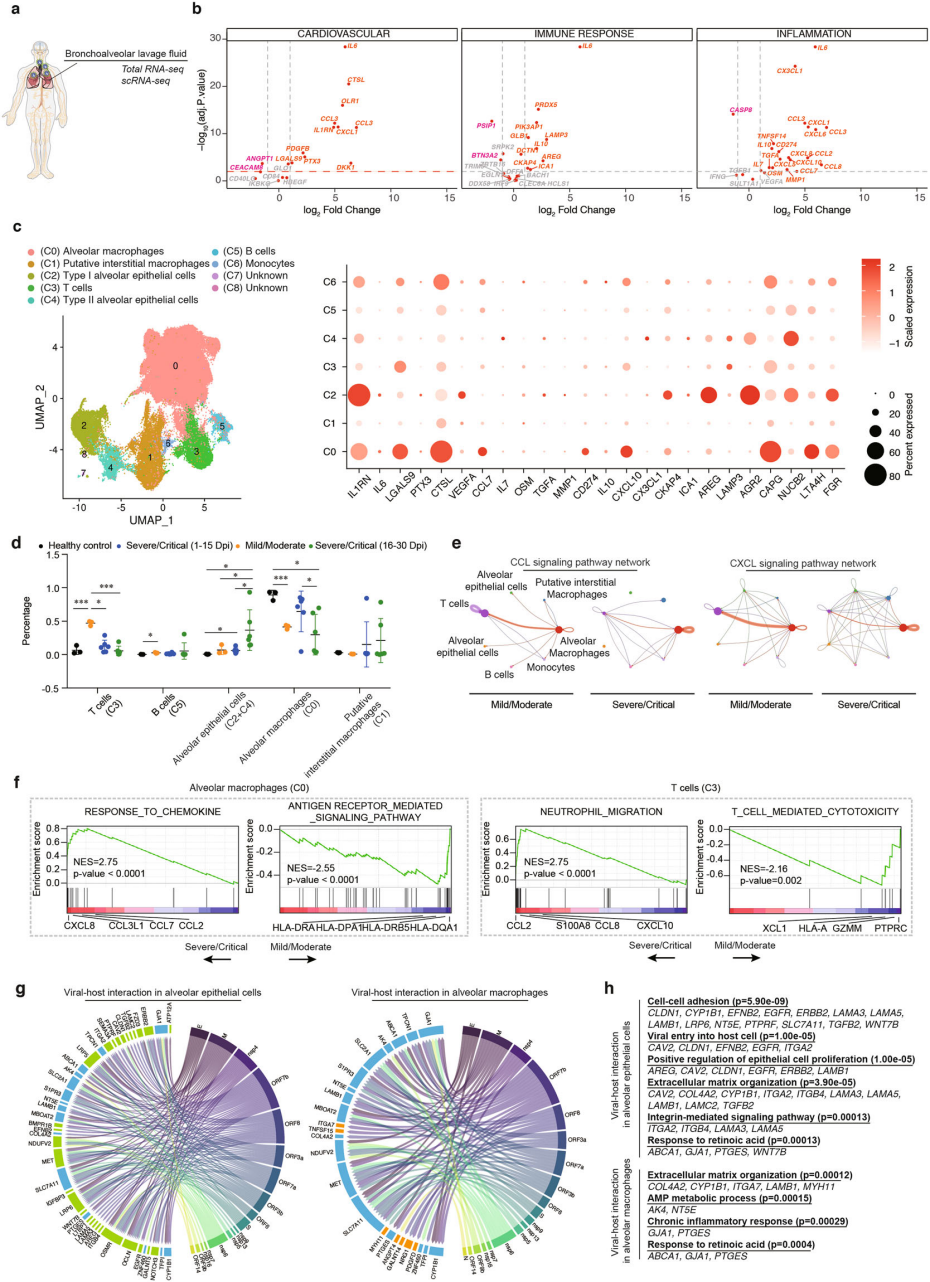
Transcriptome analysis of BALF samples in severe/critical COVID-19. **a** Schematic illustration of experiments. **b** Differential gene expression analysis using normalized RNA-seq data of BALF and peripheral blood mononuclear cell (PBMC) samples of COVID-19 patients. The fold changes of expression between BALF and PBMC and adjusted *P* values were plotted. Genes expressed at higher levels in BALF are shown in red; genes expressed at lower levels in BALF are shown in cyan. The expression matrices of PBMC and BALF are shown in Supplementary Table S4. **c** ScRNA-seq analysis of the BALF samples of COVID-19. U-map clustering of cells is shown on the left. Dot plot of genes encoding proteins elevated in COVID-19 is shown on the right. Color represents relative expression. **d** Relative fraction of T cells, plasma cells, macrophages, and alveolar epithelial cells in BALF of healthy control and COVID-19 samples. Patient samples are divided into three groups (mild/moderate, severe/critical at 1–15 dpi, severe/critical at 16–30 dpi). **e** Cell–cell communication plots of signaling networks in the BALF of mild/moderate and severe/critical COVID-19. The CCL and CXCL signaling pathways between different cell types are shown. **f** Gene Set Enrichment Analysis of genes differentially expressed between mild/moderate and severe/critical COVID-19 in T cells, plasma cells, macrophages, and alveolar epithelial cells. **g** SARS-CoV-2–host interaction maps in alveolar macrophages and alveolar epithelial cells. On each map, viral genes are shown on the right, and host genes on the left. Lines indicate virus–host interactions derived from experimentally identified protein–protein interaction maps. **h** GO analysis of genes of virus–host interacting proteins in alveolar macrophages or alveolar epithelial cells.

To explore the source of the circulating cytokine/chemokine/secreted proteins elevated in COVID-19, we analyzed the RNA-seq data of BALF and PBMC samples of both patients and healthy donors (Fig. 5a; Supplementary Table S6). Differential gene expression analysis using normalized datasets indicated that the mRNAs encoding most serum proteins elevated in COVID-19 were expressed at higher levels in COVID-19 BALF than in COVID-19 PBMC or control/healthy BALF samples (Fig. 5b; Supplementary Fig. S9 and Table S7). For example, highly elevated mRNAs in COVID-19 BALF included *IL-6*, *CXCL1*, *IL-10*, *CXCL6*, *CCL20*, *CCL3*, *MMP1*, *CX3CL1*, and *AREG* (Fig. 5b). By contrast, the majority of factors related to organ damages were expressed at similar levels in the BALF and PBMC of both COVID-19 and healthy controls (Supplementary Fig. S9 and Table S7). These results suggested that cytokines, particularly those associated with severity of COVID-19, were mainly derived from respiratory tract and/or the lung, whereas elevated circulating factors associated with organ damages were likely produced on site.

To determine which cell type(s) contributed to cytokine/chemokine release, we performed single-cell RNA sequencing (scRNA-seq) analysis using three BALF samples from patients with severe/critical conditions and integrated our dataset with previously published ones^51,52^ (Supplementary Table S8). Using curated cell type marker genes, we could identify four major cell clusters that respectively corresponded to putative marophages/monocytes (*CD68, CD86, CD163*, and *MRC1/CD206*), alveolar epithelial cells (*EPCAM* and *CHD1*), T cells (*CD3D*, *CD3E*, *CD8A*, and *CD8B*), and B cells (*CD79A*, *CD79B*, *IGHG1*, and *IGHG4*) (Supplementary Fig. S10). In addition, several previously published markers associated with macrophage subtypes allowed us to distinguish three potential subtypes, including the alveolar macrophage (*CXCL2* and *SIGLEC1*)^53,54^, the putative interstitial macrophages (*MIF* and *IFIT1*)^55,56^, and the monocyte (low levels of MHC II markers such as *HLA*-*DRB1*)^57^. Likewise, two distinct subtypes of alveolar epithelial cells (type I and type II) were also identified by the expression of *P2RX4* (type I), *P2RX7* (type I), and *ACE2* (type II) (Supplementary Fig. S10). We found that alveolar macrophages, alveolar epithelial cells, and monocytes in the lung expressed many genes encoding elevated proteins in circulation, such as *CCL20*, *IL1RN*, *IL-6, IL-7*, *CXCL5*, *TNFSF14*, *CXCL1, CMCL8*, *CKAP4*, *PIK3AP1*, *FGR*, and *BID* (Fig. 5c). The fraction of T-cell (mainly CD8^+^ T cell) number was significantly increased in mild/moderate but not in severe/critical COVID-19 (Fig. 5d), suggesting that T-cell response was mobilized in the former but impaired in the latter. These observations also implied that lymphocytopenia in severe/critical COVID-19 was unlikely due to massive lymphocyte infiltration into the lung. On the other hand, the fraction of alveolar epithelial cells was especially increased in severe/critical cases at a late stage, likely due to increased epithelial cell detachment from the alveoli as the disease progressed (Fig. 5d).

Using the scRNA-seq dataset, we further analyzed the cell–cell signaling networks in COVID-19. In comparison with mild/moderate cases, severe/critical ones were characterized by reduced CCL and CXCL signaling between alveolar macrophages and T cells, as well as increased alveolar macrophage self-activation (Fig. 5e). In agreement with this, in macrophages, genes related to chemokine responses were enriched in severe/critical cases, whereas genes related to antigen receptor-mediated signaling pathway were activated in mild/moderate cases; in T cells, genes related to neutrophil migration were enriched in severe/critical cases, whereas genes related to T-cell-mediated cytotoxicity were enriched in mild/moderate cases (Fig. 5f).

Several proteomic studies have recently established SARS-CoV-2 human protein–protein interacting atlases^58–61^. We prioritized the genes encoding SARS-CoV-2-interacting proteins based on their relative enrichment in the bulk RNA-seq data of patient BALF samples (those of healthy samples were used as controls). Of the top 50 hits, most were found to be expressed in the alveolar epithelial cells (mainly type II) in the COVID-19 BALF scRNA-seq data (Fig. 5g), consistent with the idea that these cells were the primary infection targets. Gene ontology (GO) terms associated with these proteins were enriched in cell–cell adhesion, viral entry into host cell, extracellular matrix organization, etc (Fig. 5h). Putative viral–host-interaction proteins were also identified in the alveolar macrophage, which might be contributed by phagocytosis or infection by SARS-CoV-2^62,63^. These genes were enriched in GO terms of extracellular matrix organization, AMP metabolic process, chronic inflammatory responses, etc (Fig. 5h). These findings add a new layer of information on how host gene expression networks respond to the infection in the bronchoalveolar microenvironment.

### Host response pathways in PBMC during infection and convalescence

**Fig. 6:**
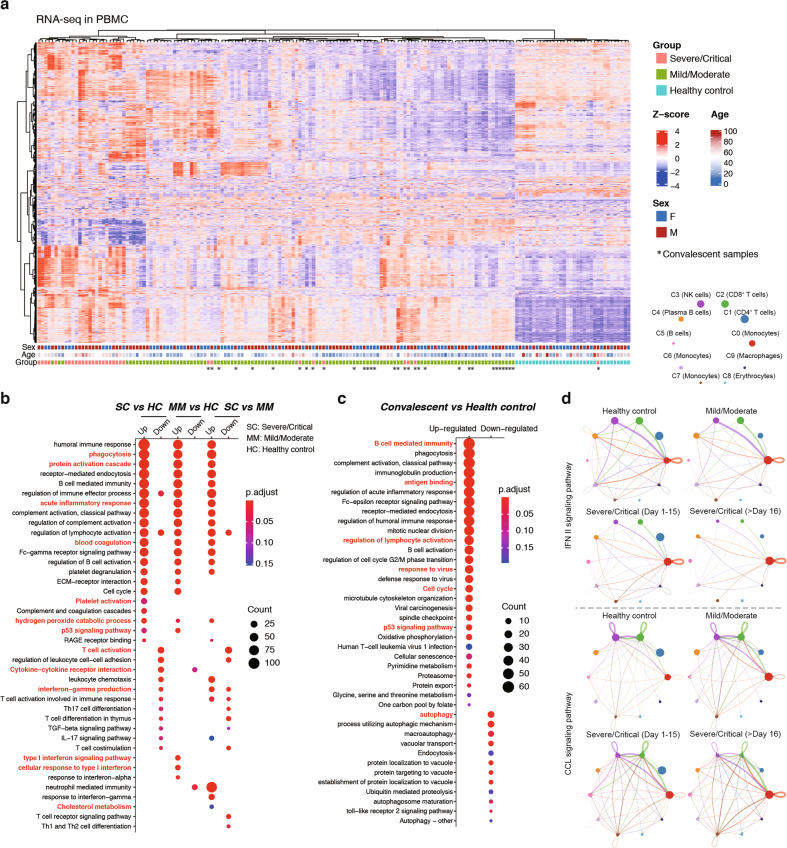
Gene regulatory networks in COVID-19 PBMC. **a** Heatmap of differentially expressed genes in the PBMC samples of COVID-19 and healthy donors. **b** GO and KEGG analyses of differentially expressed genes between sample groups. HC healthy control, SC severe/critical COVID-19, MM mild/moderate COVID-19. **c** GO and KEGG analyses of differentially expressed genes between COVID-19 convalescence samples and healthy controls. **d** Cell–cell communication maps in PBMC of healthy controls and COVID-19. The activities of IFN II and CCL signaling pathways are shown. Patient samples are divided into three groups: mild/moderate, severe/critical at 1–15 dpi, and severe/critical at 16–30 dpi. The cell types of each node are shown on the top.

Once circulating factors are released into the blood stream, they invariably exert influence on the peripheral immune cells and ensuing systemic host responses. Indeed, recent studies using COVID-19 PBMC samples have uncovered a variety of changes of the transcriptomes of immune cells among patients^64,65^. To determine how such changes occurred in our patient cohort, we performed RNA-seq using PBMC samples of 110 COVID-19 cases at infectious stages (87 samples from 81 mild/moderate cases; 23 samples from 21 severe/critical cases), 32 samples of convalescence cases (23 samples from 23 mild/moderate convalescence cases; 9 samples from 9 severe/critical convalescence cases), and 33 healthy control samples (Fig. 6a). As expected, unsupervised clustering of these data grouped the samples in accordance with disease conditions (Fig. 6a; Supplementary Table S9). We then performed differential gene expression analysis between sample groups. Compared to healthy controls, genes upregulated in COVID-19 during infectious stages were significantly associated with phagocytosis, acute inflammatory response, blood coagulation, platelet degranulation, cell cycle, p53 signaling pathway, and RAGE receptor binding (Fig. 6b). Compared to mild/moderate cases, severe/critical ones showed a significant increase of genes associated with neutrophil-mediated immunity, phagocytosis, blood coagulation, regulation of cytokine secretion, and platelet degranulation; by contrast, those downregulated in severe/critical cases were associated with T-cell activation, Th17 cell differentiation, and TGF-beta signaling pathway (Fig. 6b). Furthermore, many genes were differentially expressed between convalescent COVID-19 and healthy control samples. In particular, genes upregulated in convalescent samples were associated with activated B-cell mediated immunity, antigen binding, response to virus, which were indicative of active humoral immune responses. The convalescent COVID-19 samples also included the upregulated TP53 pathway genes and downregulated autophagy genes (Fig. 6c; Supplementary Table S10), which might reflect possible repair mechanisms.

As mentioned earlier, the alveolar microenvironment was characterized by abnormal macrophage self-activation and reduced macrophage T-cell communication in severe/critical COVID-19 (Fig. 5e). To determine if a similar situation existed in the peripheral blood, we performed the same cell–cell interaction analysis using recently published scRNA-seq datasets of COVID-19 PBMCs^66,67^. As shown in Fig. 6d and Supplementary Fig. S11a, the main difference between healthy control and different patient groups (classified by severity and dpi) resided in signaling activities between monocytes and lymphocytes (e.g., T, NK, B cells). For example, the type II IFN signaling activity between monocytes and NK/CD8^+^ T cells was high in mild/moderate but low in severe/critical COVID-19, especially at a late stage (> 16 dpi). Conversely, self-activating CCL and CXCL pathway activities in monocytes were higher in severe/critical cases than in mild/moderates ones or control samples (Fig. 6d; Supplementary Fig. S11b). Thus, CCL/CXCL signaling was involved in the over-activation of both alveolar macrophage and peripheral monocytes, and the communications of these two cell types with lymphocytes were, respectively, defective in the lung and the peripheral blood.

### TP53 is alternatively spliced in COVID-19 PBMC

**Fig. 7:**
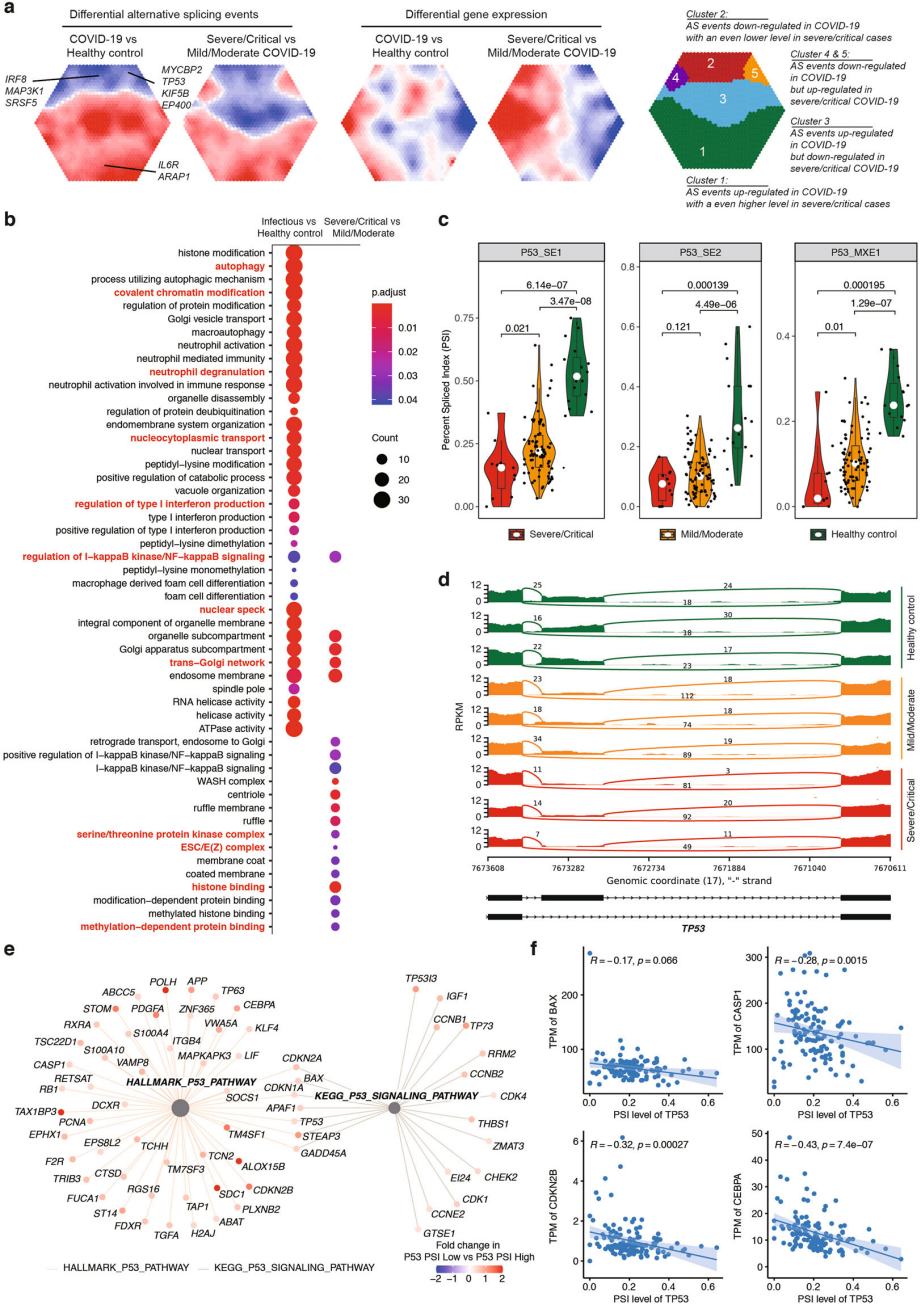
Alternative splicing in COVID-19 PBMCs. **a** Self-organizing map of differential alternative splicing events in COVID-19 vs healthy control, or severe/critical vs mild/moderate COVID-19. The differential percent spliced index (PSI) was used for the self-organizing map (Left panel). Differential gene expressions were used for self-organizing map in the middle panel. Annotations of clusters were plotted in the right panel. **b** GO analysis of differentially spliced genes. **c** Differential splicing events of *TP53*. The percent-spliced index (PSI) scores of these events in healthy and COVID-19 samples were plotted. **d** Representative illustration of the alternative splicing events of *TP53* in healthy and COVID-19 samples. **e** TP53 pathway activity in COVID-19. **f** Correlation between *TP53* alternative splicing events and P53 pathway activity. Transcript per million (TPM) values of *BAX*, *CASP1*, *CDKN2B*, and *CEBPA* were shown. The PSI score of *TP53* was computed by KEGG Gene Set Enrichment Analysis.

Besides transcriptional regulation, AS has been suggested as a mechanism by which SARS-CoV-2 affects the gene activities in host cells^68^. Therefore, we performed a comprehensive analysis of AS events in the 110 COVID-19 PBMCs RNA-seq dataset at infectious stages (87 samples from 81 mild/moderate cases; 23 samples from 21 severe/critical cases) and 33 healthy control samples, including alternative 3′ splice site (A3SS), alternative 5′ splice site (A5SS), mutually exclusive exons (MXE), retained intron (RI), and skipped exon (SE). In total, 10,100 AS events in 3300 genes were found to differ between COVID-19 and healthy controls. Among these, 2924 AS events were significantly different between mild/moderate and severe/critical COVID-19 (Fig. 7a; Supplementary Table S11). GO analysis indicated that genes with increased AS scores were significantly associated with autophagy, regulation of type I IFN response, and neutrophil degranulation (Fig. 7b; Supplementary Table S12). For most of these genes, the total transcript levels were upregulated in COVID-19 samples and positively correlated with disease severity (Fig. 7b). The AS scores also significantly differed between severe/critical and mild/moderate COVID-19, despite little difference in their total transcript levels (Fig. 7b). These results indicate widespread AS of genes upon SARS-CoV-2 infection.

Interestingly, the AS scores of three isoforms of TP53 (P53_SE1, P53_SE2, and P53_MXE1) were dramatically reduced in both mild/moderate and severe/critical COVID-19 samples, and the AS scores in severe/critical cases were even lower than those of mild/moderate ones (Fig. 7c). As shown in Fig. 7d, COVID-19 PBMCs preferentially expressed TP53 isoforms skipping the ninth exon that contains a stop codon, and thus produced a full-length TP53 with complete C-terminal regions essential for its DNA-binding activity^69^. Indeed, the TP53 pathway activity in patient RNA-seq datasets was siginificantly increased (Fig. 7e) and the AS scores negatively correlated with the expression of canonical TP53-target genes (Fig. 7f). Further studies may be required to functionally evaluate the role of these isoforms in COVID-19 in vitro and/or in vivo.

## Discussion

Thus far, a large number of studies have described the symptoms of severe and critical COVID-19, including ARDS, lymphocytopenia, CRS, aberrant coagulation, and multiorgan damages^17,20,24^. However, detailed analysis of how these events unfold during various stages of COVID-19 is still lacking and, as a result, the dynamics of systemic host response to SARS-CoV-2 remain unclear. Here, using an integrative analysis of clinical laboratory tests, transcriptomes of BALF and PBMC, and targeted proteomes of patient sera, we have investigated the dynamic changes of the major phenotypic features of COVID-19 during the first month post-illness onset. The results show that the earliest indicators of disease deterioration toward severe/critical conditions are lymphocytopenia and elevation of markers of kidney injuries, which precede abnormal cytokine releases and elevation of markers of ARDS, coagulation, and cardiovascular/liver damage. One implication of these observations relates to the role of CRS in COVID-19^21,70^. That is, although CRS has been proposed as a key driver of several symptoms of severe COVID-19, the relative lag of cytokine increases with respect to acute loss of lymphocytes in patient blood suggests that these cytokines might not be directly responsible for early lymphocytopenia. In consistence with this, proliferation of lymphocytes from healthy blood samples was promoted, rather than suppressed, upon treatment with most cytokines elevated in severe COVID-19. On the other hand, our study provides evidence for aberrantly increased cortisol in patient serum as one of the probable causes of lymphocytopenia. It is noteworthy that elevated cortisol levels have been shown to correlate with mortality rate in severe/critical COVID-19^71,72^, and hydrocortisone can regulate the kinetics of human lymphocytes, especially at the time of stress^73,74^. Nevertheless, although several clinical trials have adopted corticosteroids in treating severe/critical COVID-19, the efficacy of such therapy remains controversial^75,75–80^. Further studies are warranted to determine the role of cortisol and cytokines in lymphocytopenia in order to improve the efficiency of pre-empting the patients from developing severe conditions.

Through comprehensive measurement of cytokines, chemokines, and secreted proteins, we have characterized many factors specifically enriched in the serum of severe COVID-19 patients. Our study confirmed that, in mild/moderate cases, a “core” COVID-19 signature characterized by elevated levels of several cytokines (e.g., IL-1α, IL-1β, IFN-α, IL-17A, and IL-12), which was first identified in a longitudinal study of immune responses in 113 COVID-19 patients by Lucas et al.^81^. Moreover, we characterized the dynamics of cytokine releases in severe/critical COVID-19, including the lack of IFN-α and IL-17E at the early days after disease onset, and distinct fluctuations of two groups of cytokines, respectively, represented by IL-6 at 5–20 dpi and IL-8 at 16–30 dpi. In addition, we have identified a large number of signaling molecules with various roles in modulating the proliferation and activation of immune cells, including TNFRSF1A and TNFRSF1B. Combining these proteomic analyses with differential gene expression analyses between severe/critical and mild/moderate COVID-19 cases, we were able to construct a network of interconnecting genes/proteins and characterize the variation of the network components at different stages of COVID-19 (e.g., during infection and convalescence) (Supplementary Fig. S12a). This network may be useful to explore potential therapeutics that might influence its activities (Supplementary Fig. S12b–d).

Because the clinical presentation of COVID-19 starts from lung infection and spreads to other organs by circulatory immune responses and secondary infection, we have focused our analysis on the signaling activities in the alveolar microenvironment and peripheral blood. Based on comparative RNA-seq analyses at bulk and single-cell levels, three general themes can be distinguished. First, in the BALF of COVID-19, the number of T lymphocytes are increased in mild/moderate cases but remain low in severe/critical cases. Such ineffective T-cell response is associated with over self-activation of the alveolar macrophage and low CCL/CXCL signaling between alveolar macrophage and T cells in the alveoli. A similar reduction of CCL/CXCL communications also exists between blood monocytes and T cells. These findings suggest that weakened cellular immune responses underline the severity of COVID-19. Second, in severe COVID-19 patients, genes encoding most elevated cytokines, chemokines, and signaling pathway ligands are specifically expressed at much higher levels in the BALF than in the PBMCs, suggesting that increased expression of these factors originated from the alveolar microenvironment and then secreted into the circulatory system. Third, COVID-19 peripheral blood is characterized by a variety of signaling pathway activities indicative of both disease severity and stage. In particular, those highly enriched in severe COVID-19 during the infection phase are primarily associated with innate immune responses and organ damages, whereas those enriched in convalescence samples are primarily associated with B-cell-mediated immune responses and possible repair mechanisms. Of particular interest is the enhanced AS events generating the full-length TP53 in COVID-19 PBMC, though its biological importance needs further exploration. The distinct changes of these gene activities as a result of both transcriptional regulation and AS suggest that versatile mechanisms are involved in host cell responses to SARS-CoV-2 infection.

## Materials and methods

### Ethics statement

This study was approved by the Shanghai Public Health Clinical Center Ethics Committee. Informed consents had been obtained from all the enrolled patients.

### Clinical cases and laboratory tests

A total of 963 COVID-19 cases, who were admitted to Shanghai Public Health Clinical Center from 21st January to 17th October of 2020, and 36 healthy donors were enrolled in this study. The COVID-19 cases were diagnosed as mild/moderate or severe/critical according to the Diagnosis and Treatment Protocol for COVID-19 Patients (Tentative 8th Edition) (http://en.nhc.gov.cn/2020-09/07/c_81565.htm). The basic demographic and clinical features are shown in Supplementary Table S1. All enrolled COVID-19 cases were treated according to the instruction of 8th edition of Diagnosis and Treatment Protocol for COVID-19 Patients in China. In addition, 14 patients received immunomodulatory treatment (7/33 severe/critical cases and 6/930 mild/moderate cases received methylprednisolone treatment; 1/33 severe case received the Trastuzumab treatment), for whom only samples collected before immunomodulatory therapy were used for cytokine/chemokine profiling experiments.

Routine blood tests, including lymphocyte counts, lymphocyte percent, monocyte counts, monocyte percent, neutrophil counts, neutrophil percent, platelet counts, red blood cell count (RBC), HCT, Hb, mean corpuscular hemoglobin (MCH), MCH concentration, mean corpuscular volume, and mean platelet volume, were performed using the Sysmex XN-1000 Hematology Analyzer. Coagulation-related markers, including APTT, D-D, FDP, INR, and fibrinogen, were detected with the STA-R MAX coagulation analyzer. Subsets of lymphocytes were labeled with the BD Multitest™ 6-color TBNK and detected by the BD FACSCanto™. Biochemical analyses including, α1-acid glycoprotein, ALB, ALP, ALT, aspartate aminotransferase (AST), cholinesterase, CK, CK-MB, Cr, indirect bilirubin, direct bilirubin, total bile acid, total bilirubin, lactic acid, lactic acid dehydrogenase, prealbumin, total protein, apoA1, apoB, apoE, cystatin C, glutathione reductase, homocysteine, HDL, low-density lipoprotein, lipoprotein, retinol-binding protein, total cholesterol, and triacylglycerol were performed by ACCELERATOR a3600/ARCHITECT c16000 Automatic biochemistry analyzer (Abbott Diagnostics).

The samples were used for these experiments based on availability, and whenever possible, each sample was used for multiple tests. No data or samples were excluded during data analysis to minimize sample selection bias.

### Isolation of mononuclear cells from peripheral blood and BALF

The mononuclear cells of the peripheral blood were separated using Ficoll-Paque density gradient centrifugation. Briefly, 4-mL peripheral blood in anticoagulation tubes was used for separation. Five milliliter of Ficoll-Paque PLUS reagent (GE Healthcare) was first added in a 15-mL Conical Sterile Polypropylene Centrifuge Tubes, the 4-mL peripheral blood was then dropwise added into the tube. The tube was centrifuged at 2000 rpm for 20 min. The middle layer of mononuclear cells was collected and washed twice in PBS. Cells from the BALF samples were by centrifuging the samples at 2000 rpm for 10 min.

### Assay of cytokine/chemokines on peripheral blood mononuclear cells

PBMCs isolated from the peripheral blood of healthy donors were cultured in RMPI 1640 supplemented with 10% Human Serum AB (GEMINI). Different cytokines/chemokines were added to culture media for 36 h, after which the counts of CD4^+^, CD8^+^, CD19^+^, and CD56^+^ cells were determined by the BD LSRFortessa™ X-20. The cytokines/chemokines used were: recombinant Human IL-6 (200 pg/mL), recombinant Human IL-8 (200 pg/L), recombinant Human IL-10 (400 pg/mL), recombinant Human IL-15 (100 pg/mL), recombinant Human CXCL10 (1600 pg/mL), recombinant Human GM-CSF (400 pg/mL), recombinant Human IL-18 (1500 pg/mL), recombinant Human IL-1RA (100 pg/mL), recombinant Human PD-L1 (1500 pg/mL), recombinant Human CX3CL1 (1000 pg/mL), recombinant Human TNFRSF1B (5000 pg/mL), recombinant Human TNFRSF1A (8000 pg/mL).

### Luminex-based cytokine/chemokine detection

Magnetic Luminex Assay kits were purchased from R&D Systems (catalog number: FCSTM18-45 and LXSAHM-17). The tested cytokines/chemokines were as follows: CCL2 (MCP-1), CCL3 (MIP-1α), CCL4 (MIP-1β), CCL5 (RANTES), CCL11 (Eotaxin), CCL20 (MIP-3α), CCL19 (MIP-3β), CX3CL1 (Fractalkine), CXCL1 (GRO-α), CXCL2 (GRO-β), CXCL10 (IP-10), IL-1α, IL-1β, IL-1ra, IL-2, IL-3, IL-4, IL-5, IL-6, IL-7, IL-8, IL-10, IL-12 p70, IL-13, IL-15, IL-17A, IL-25 (IL-17E), IL-33, EGF, FGF basic, VEGF, PDGF-AA, PDGF-AB/BB, TGF-α, G-CSF, GM-CSF, PD-L1 (B7-H1), CD40 Ligand, FLT-3Ligand, IFN-α, IFN-β, IFN-γ, TNF-α, TRAIL, Granzyme B, IL-2Rα (CD25), CXCL9 (MIG), gp130, IL-1R II, IL-6Rα, RAGE, TNF RI, TNF RII, angiopoietin 1, angiopoietin 2, IL-18, CCL22, BCMA, VEGF-R1, VEGF-R2, VEGF-R3, and ICAM-1. Serum samples cryopreserved at −80 °C were thawed and analyzed according to the manufacturers’ instructions. Assay plates were measured by the Luminex X-200 instrument and analyzed by MILLIPLEX Analyst using cubic curve-fit.

### O-link proximity extension assay

All serum samples of COVID-19 patients and healthy donors were heat-inactivated at 56 °C for 15 min to inactivate the virus. A total of 345 proteins in fours O-link panels, including immune response, cardiovascular II, organ damage, and inflammation, were tested according to the manufacturer’s instructions (O-link, Uppsala, Sweden). Briefly, 1-μL serum was incubated with a set of probes, and when a matched pair of antibody probes bind to their target protein, corresponding DNA labels are brought into proximity, and a proximity-dependent DNA polymerization forms a PCR target sequence. The newly formed DNA molecule is then amplified and quantified by real-time PCR using the Fluidigm Biomark™ HD system.

Raw image files of each plate are imported into the Fluidigm Real-Time PCR Analysis software and heat maps of Ct values were exported for downstream preprocessing using O-link NPX Manager. O-link data are represented by an arbitrary unit called Normalized Protein eXpression (NPX) on a log2 scale where a larger number represents a higher protein concentration in the sample. Hence, an estimate of 1 would indicate a doubling of concentration on the unknown original scale. The NPX values are obtained by normalizing Ct values against extension controls, inter-plate controls, and a correction factor. Proteins with a missing frequency > 50% across all samples were filtered, leaving 315 proteins for the downstream statistical analysis.

*P* values were calculated using the two-sided Wilcoxon Rank Sum test. False discovery rate (FDR) was controlled by correcting the *P* values according to the Benjamini–Hochberg procedure (FDR of 0.05). The difference between the average NPX values of the two sets of samples was used as the log2 fold change of the protein concentration. Proteins with an adjusted *P* values < 0.05 and an absolute estimate > 1.5 were considered significantly differential O-link biomarkers.

### RNA-seq

Cells were resuspension in 1-mL TRIzol™ Reagent. RNAs were extracted according to the manufacturer’s instruction (Invitrogen, USA). A total of 1-μg RNA was used for RNA-seq library construction with the KAPA RNA HyperPrep Kit with RiboErase (HMR) (For Illumina^®^ Platforms). A total of 125 PBMCs and 5 BALF RNA-seq libraries were generated and sequenced by the Illumina^®^ Nova-seq 6000 platform. In addition, RNA-seq data of one public dataset GSE152418 from the GEO database were also included in this study. The salmon program (version 1.2.1)^82^ was used to quantify the expression of RNA-seq transcripts using human GRCh38 as reference. Batch effects between GSE152418 and our data were removed by the limma R package (version 3.44.3)^83^. Differentially expressed genes between groups were determined using the DESeq2 package (version 1.20.0)^84^.

### scRNA-seq

Cells from three BALF samples were collected. Dead cells were removed before scRNA-seq experiments. Single-cell libraries were constructed according to the manufacture’s instruction of GEXSCOPE^®^ Single Cell Transcriptome Reagents. The libraries were sequenced on the Illumina Nova-seq 6000 platform. The expression matrix was generated using the celescope software. For BALF scRNA-seq analysis, we combined our dataset and 15 other BALF scRNA-seq datasets in the GEO database (GSE145926 and GSE147143). For PBMC scRNA-seq analysis, the published datasets from two studies were used (GSE149689 and GSE150728). Datasets were intergrated and clustered using the Seurat softwere^85^. Each clusters were annotated by cell-specific markers, including *CD14*, *CD68*, *CD86*, *CD3D*, *CD3E*, *EPCAM*, *MIF*, *SEGLEC1*, *CXCL2*, *CD163*, *MRC1*, *CD79A*, *MS4A1*, *IGHG1*, *CD19*, and *HLA*-*DRB1*. Batch effects between different datasets were removed, and the data were integrated by the harmony algorithm^86^. Dotplots of gene expression, the differential of gene expression among different groups on distinct cell types were generated by the Seurat R package^87^. Cell–cell signaling pathways were analyzed using CellChat^88^.

### AS analysis

Raw sequencing reads were mapped to the human reference genome (hg38) by the STAR algorithms^89^. AS events were detected using rMATS^90^. Five main types of AS events, including SEs, A5SSs, A3SSs, MXE, and RIs were examined. The Percent-Spliced-In matrix was extracted and imported into limma package^83^ for calculating significantly different AS events after filtering out commonality (events detected in > 80% of all samples). Sashimi plots were used for better visualization of AS events.

### Annotation of gene functions

The GO analysis^91^, Kyoto Encyclopedia of Genes and Genomes analysis^92^, and Gene Set Enrichment Analysis were performed using the clusterProfiler (version 3.16.1)^93^. The results were visualized by the ggplot2 (version 3.3.2) and ComplexHeatmap (version 2.4.3) R packages^94^. Genes and samples were clustered using the ward.D or the ward.D2 method, and distance was calculated based on the Euclidean or Pearson’s correlation coefficient in the gplots package (version 3.0.1.1)^95^.

### Mice experiments

The effect of cortisol on murine PBMCs was tested in 12 male C57/BJ6 mice (6–8 weeks old). The mice were randomly divided into two groups (6 per group) and subcutaneously injected with 0.1 mg of cortisol (dissolved in 100-μL PBS) or PBS alone. At 36 h after injection, blood was collected from each mouse, the RBCs were lysed, and the relative numbers of CD4^+^, CD8^+^, CD19^+^, and Mac1^+^ cells were determined by flow cytometry using the BD LSRFortessa™ X-20 Cell Analyzer.

## Supplementary information

Supplementary Figures and Figure Legends

Supplementary Table S1

Supplementary Table S2

Supplementary Table S3

Supplementary Table S4

Supplementary Table S5

Supplementary Table S6

Supplementary Table S7

Supplementary Table S8

Supplementary Table S9

Supplementary Table S10

Supplementary Table S11

Supplementary Table S12

## Data Availability

The RNA-seq data and scRNA-seq data are available at https://dds.linc.org.cn/pub/PRJ20201216. Additional information and request for the material and method detailed should be directed and will be fulfilled by the Lead Contact.
