# Supplementary material for: Integrating longitudinal clinical laboratory tests with targeted proteomic and transcriptomic analyses reveal the landscape of host responses in COVID-19
Source: Cell Discov. 2021 Jun 8;7:42. doi: 10.1038/s41421-021-00274-1 (PMC8185699; doi:10.1038/s41421-021-00274-1)
Supplement: Supplementary file 1 — Supplementary Figures and Figure Legends [file 41421_2021_274_MOESM1_ESM.pdf]

Supplementary Information

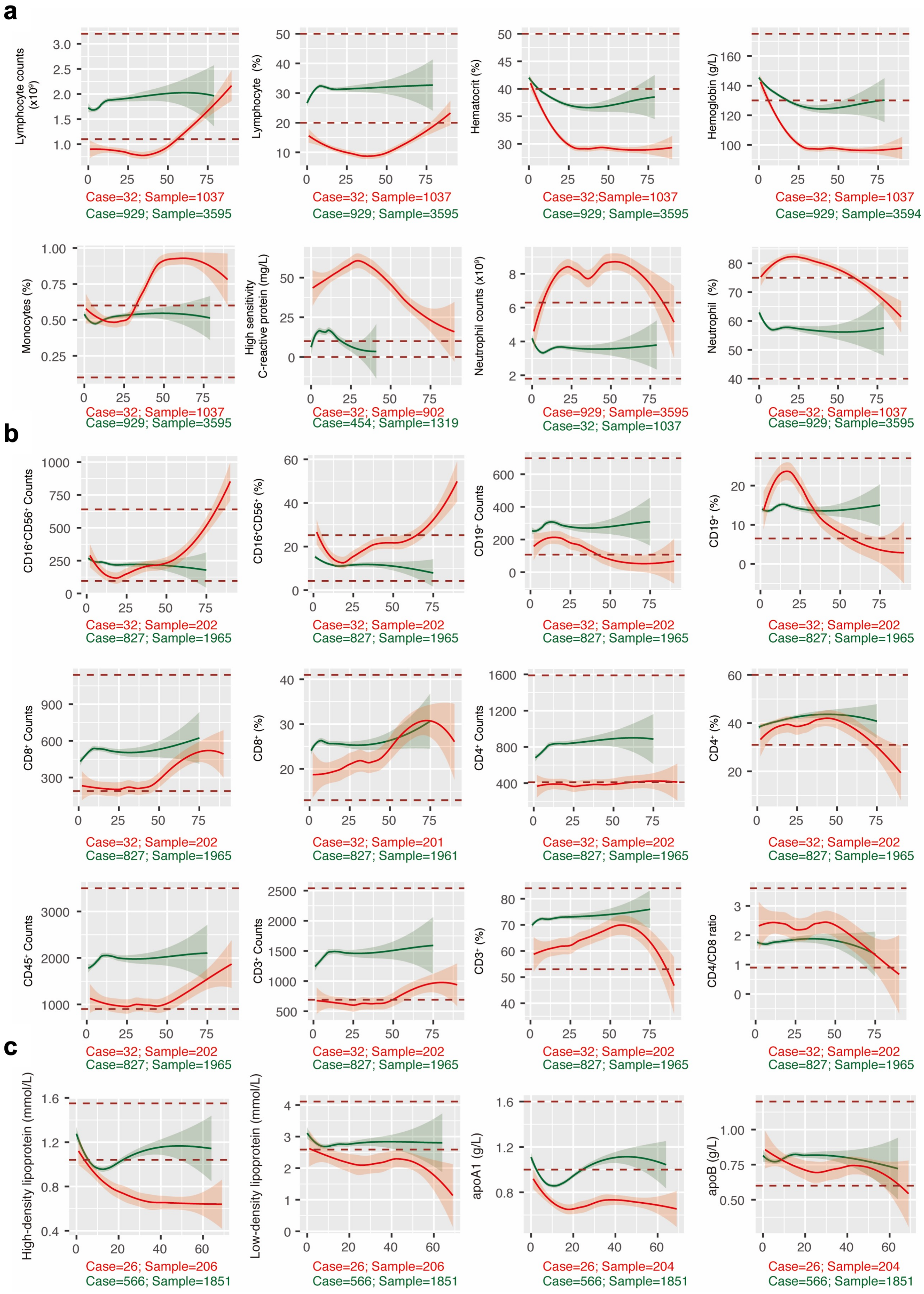

Supplementary Fig. S1. Long-term observation of routine blood test, lymphocytes, and blood lipid tests post-SARS-COV-2 infection. Related to Fig. 1.

(**a**) The lymphocytes, neutrophils, monocytes turned to normal while the red blood cells were still abnormal in the recovery stage of severe/critical COVID-19. The levels of lymphocyte counts, lymphocyte percent, neutrophil counts, neutrophil percent, monocyte counts, monocyte percent, hematocrit, the hemoglobin within 3 months post-infection were plotted. The red color represents the severe/critical COVID-19 and the green color represents the mild/moderate COVID-19. The normal range of each test was plotted with dashed lines. The x-axis represents the days post illness onset (Dpi). (**b**) The $CD4^+$, $CD8^+$, $CD19^+$ lymphocytes restored to the normal range but the $CD16^+CD56^+$ lymphocytes appeared to be elevated at the recovery stage. (**c**) The high-density lipoprotein, low-density lipoprotein, apoA1 were decreased and remain a low level on the recovery stage.

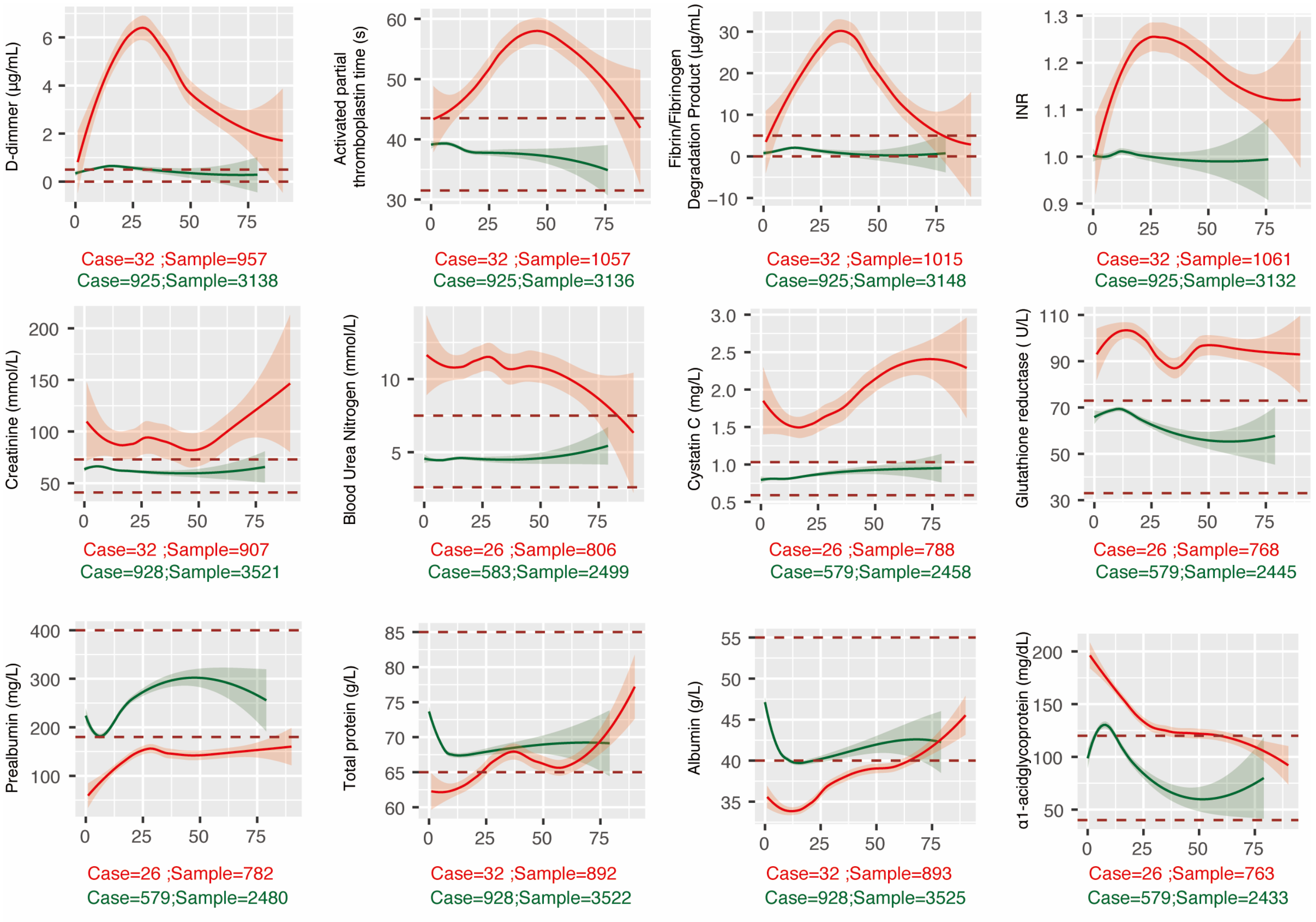

Supplementary Fig. S2. Long-term observation of coagulation, multi-organ damage related test post-SARS-COV-2 infection. Related to Fig. 1.

The coagulation turned to normal in the recovery stage of severe/critical COVID-19. The coagulation related markers were elevated from dpi 1 to dpi 25~50 but restored to the normal range at the recovery stage. The levels of coagulation related markers, including D-dimer, activated partial thromboplastin time, Fibrin/Fibrinogen Degradation Product, and INR within 3 months post-SARS-COV-2 infection was plotted. The liver function turned to normal while the kidney remained to be injured in the recovery stage in severe/critical COVID-19.

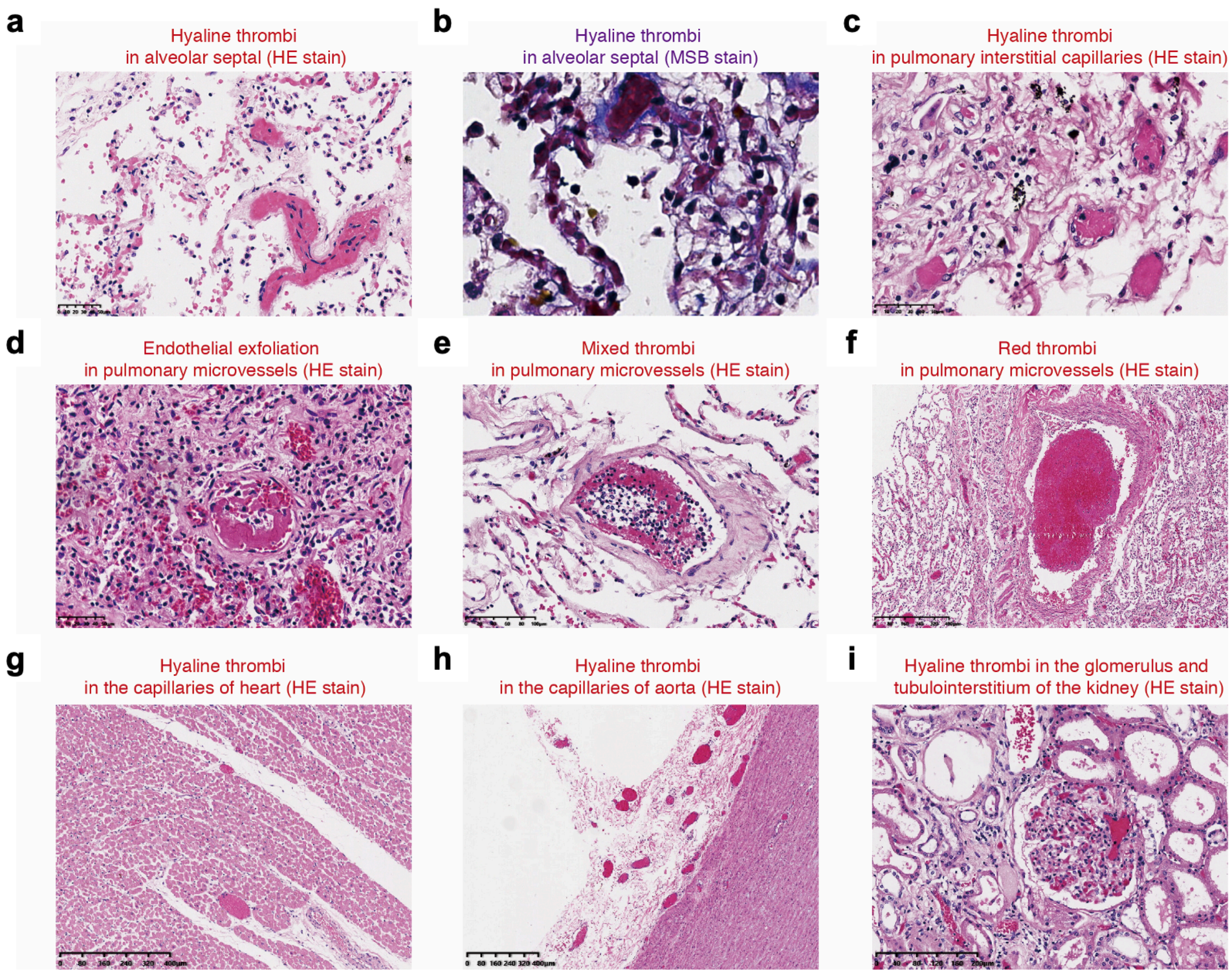

Supplementary Fig. S3. Histopathology showing intensive Thrombi in multi-organs of COVID-19 deceased patients.

Different types of thrombi, mainly mixed and hyaline thrombi, were found in pulmonary, nephrotic and myocadial tissues from all of the COVID-19 cases. Endothelial cells showed exfoliation from microvessels (**d**). The hyaline thrombi composed of fibrin and platelets existed mainly in capillaries (**a, c, g, h, i**), while the mixed one often in small veins such as venules (**e**). Red thrombi could occasionally be found in some veins (**f**), and arterial thromboembolism could be present in some cases. The hyaline thrombi were preferentially distributed in the capillaries of pulmonary interstitium and alveolar septum, especially in exudative lesions and pleural regions. Even in the pulmonary tissues with minor inflammation, hyaline thrombi could also be easily identified. Similar thrombi were examined in the myocardial interstitium, glomerular capillaries, and small veins of the kidneys (**i**). However, the incidence of thrombi in the heart and kidneys were relatively lower than that in the lung.

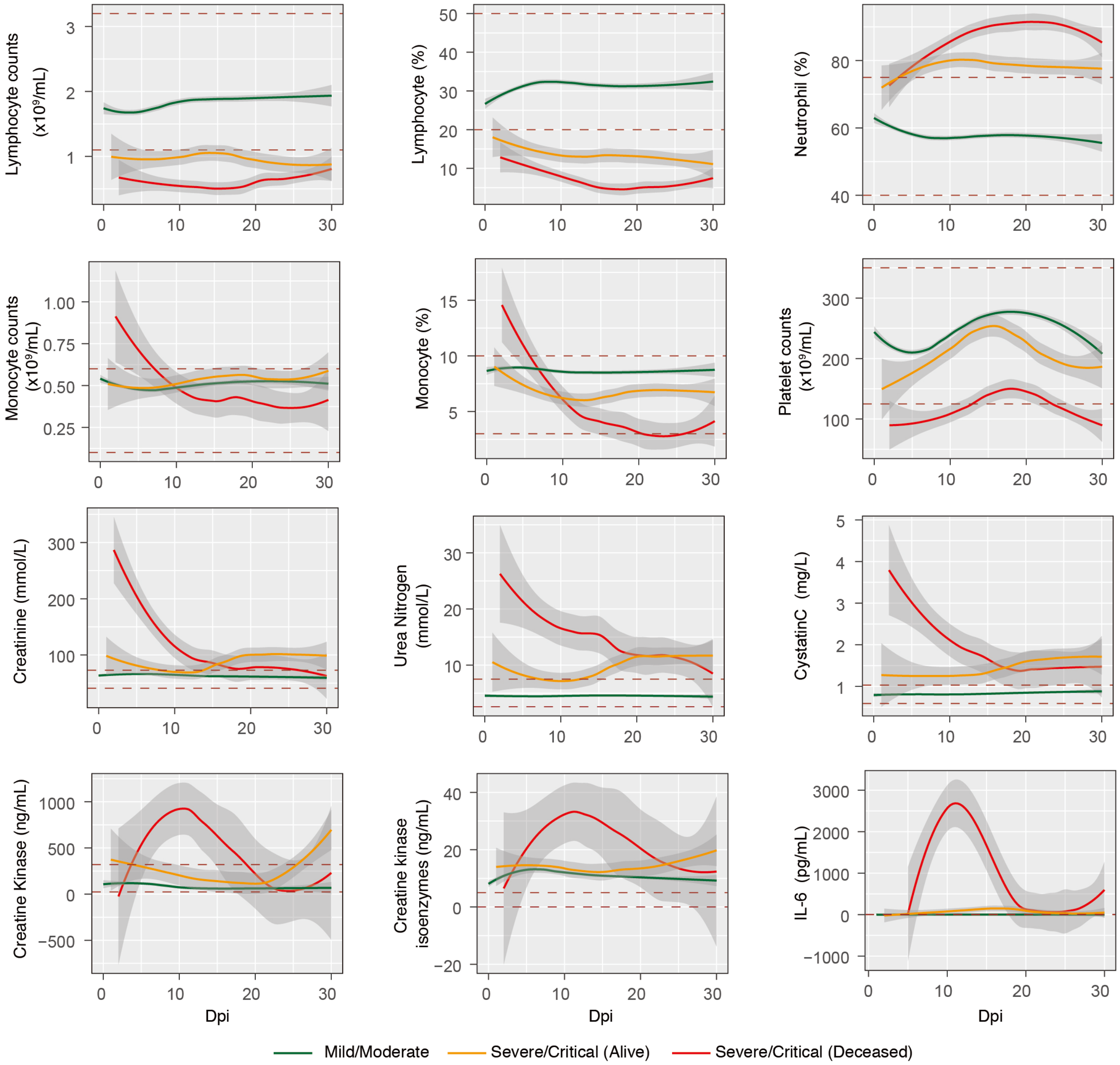

Supplementary Fig. S4. **Longitudinal analysis of laboratory tests of serum from COVID-19 patients.**

The levels of lymphocyte counts, lymphocyte ratio, neutrophil ratio, monocyte counts, monocyte ratio, platelet counts, creatinine, urea nitrogen, cystatin C, creatine kinase, creatine kinase isoenzymes, and IL-6 are shown. A total of 921 mild/moderate cases, 6 deceased severe/critical cases, and 26 severe/critical cases without LMWH treatment were used for plotting.

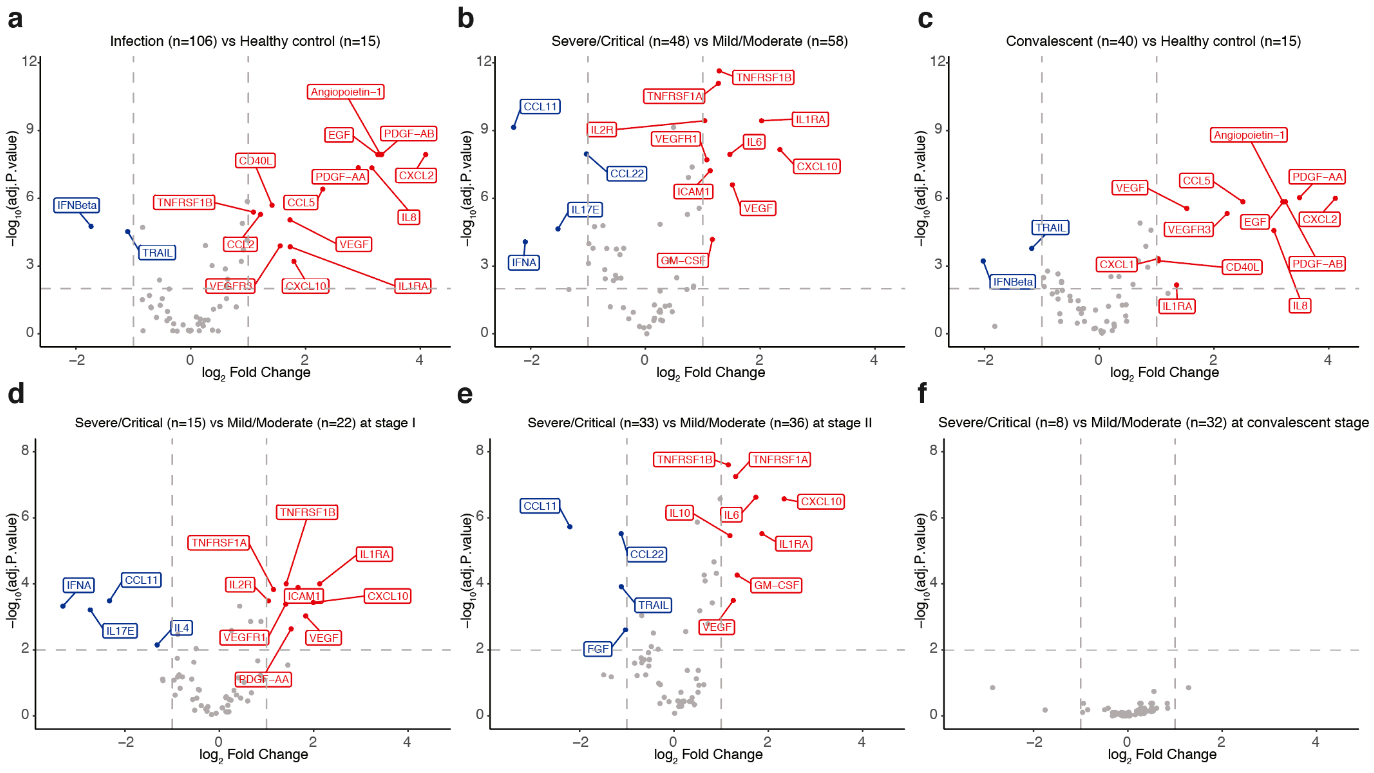

Supplementary Fig. S5. Pair-wise comparison of plasma levels of cytokines and chemokines between different groups/stages of SARS-COV-2 cases post-infection. Related to Fig. 2.

The log2 fold changes and adjusted p-values in SARS-COV-2 infection vs. healthy control were plotted. Significantly changed cytokines/chemokines were labeled.

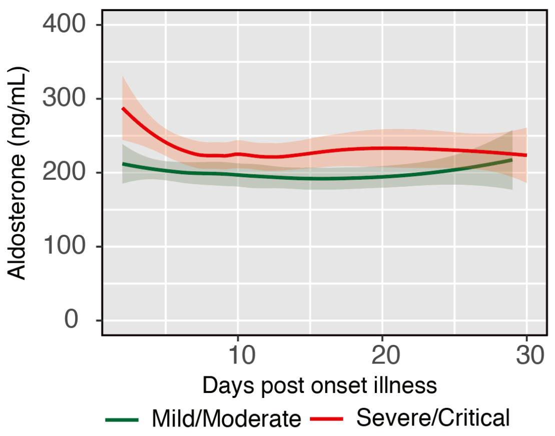

Supplementary Fig. S6. Longitudinal analysis of aldosterone levels in COVID-19 patients. Related to Fig. 3.

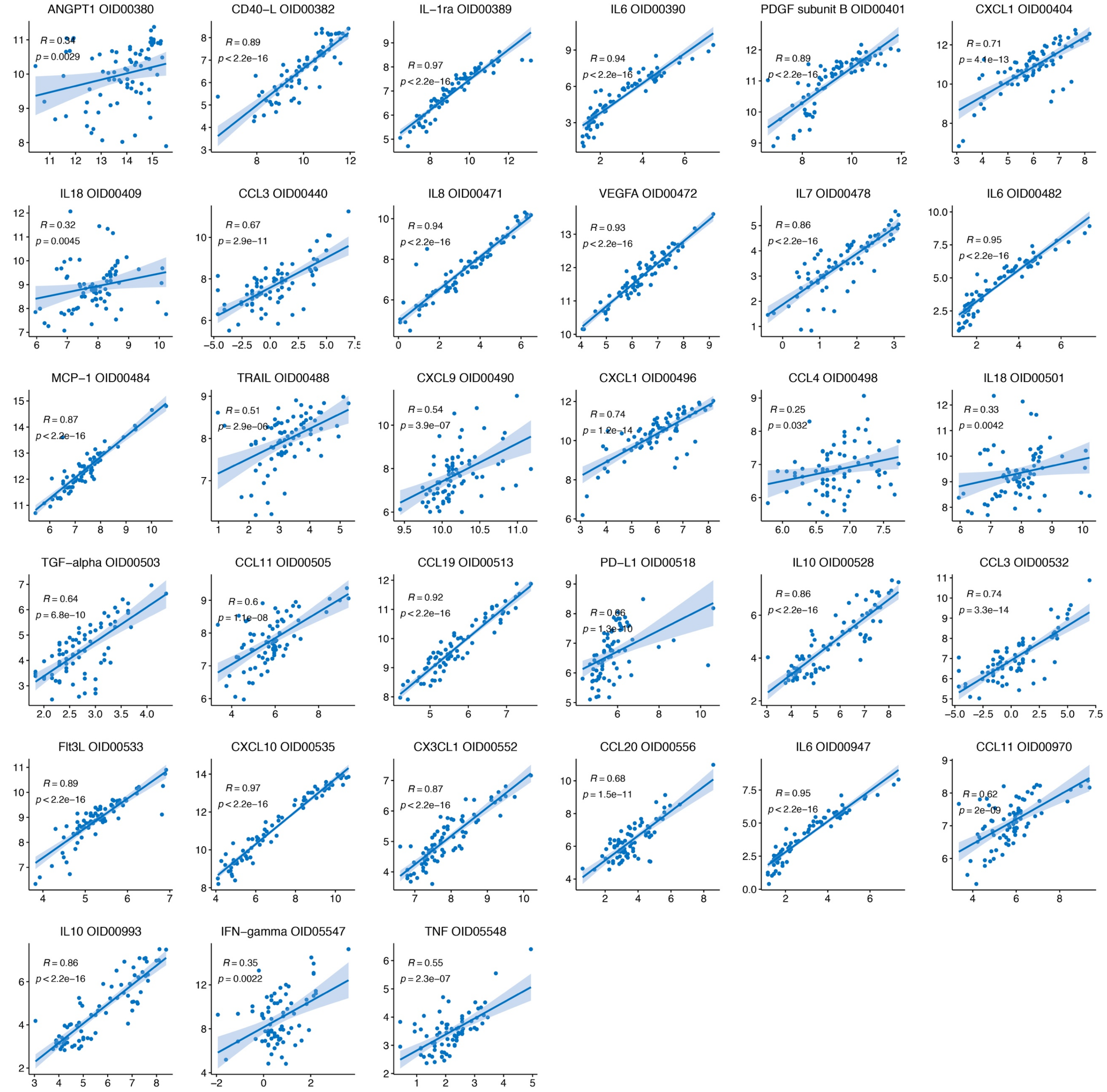

Supplementary Fig. S7. Correlation between Luminex-based cytokine/chemokine profiling and O-link based proteomics. Related to Fig. 4.

The estimated score of each cytokines/chemokine detected by O-Link (in Figure 3) and the Log2-tranfered expression of the same cytokine/chemokine detected by Luminex (in Figure 4) were plotted. The correlation score and p value were shown.

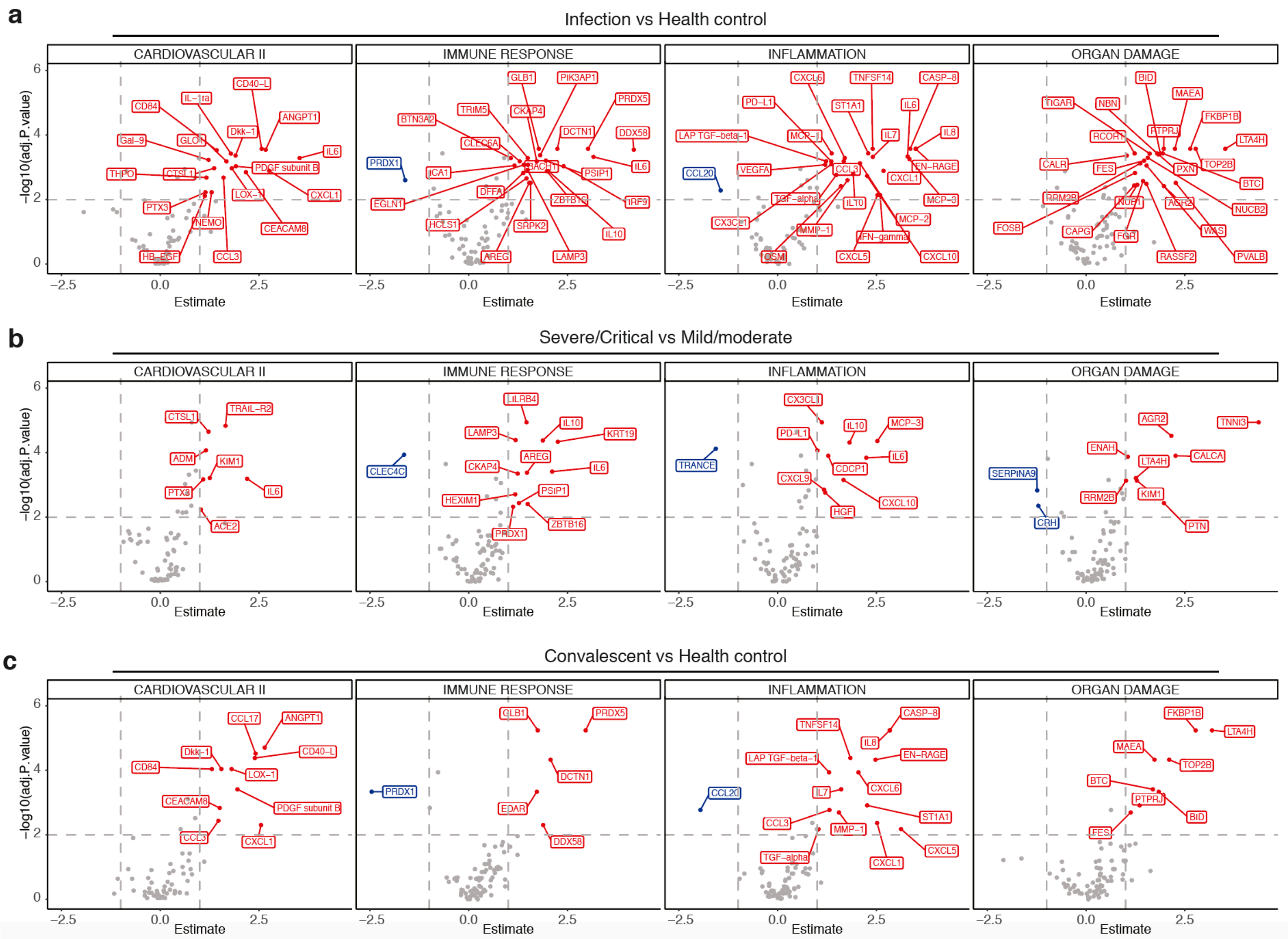

Supplementary Fig. S8. Analysis of differential plasma proteins post-SARS-COV-2 infection. Related to Fig. 4.

(**a**) Dysregulated plasma proteins post-SARS-COV-2 infection. (**b**) Plasma proteins differentially expressed in severe/critical COVID-19 as compared to mild/moderate COVID-19. (**c**) Plasma proteins differentially expressed at the convalescent stage of COVID-19 as compared to healthy control donors.

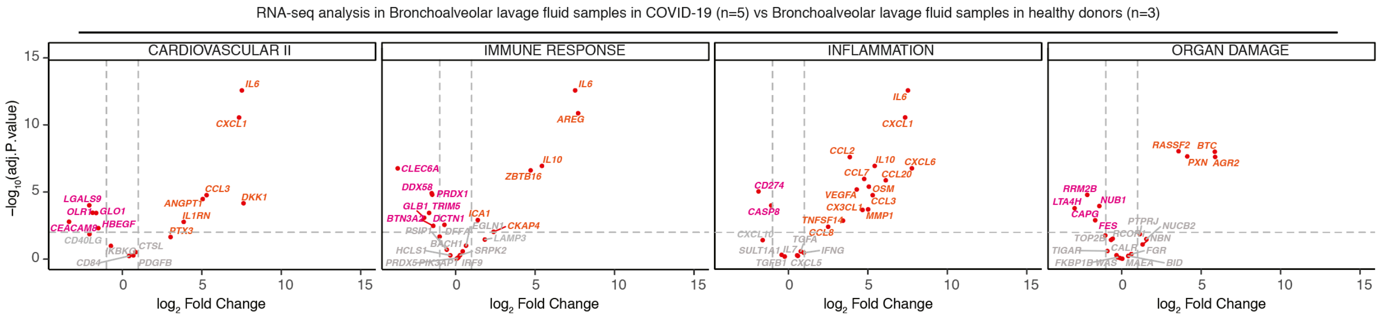

Supplementary Fig. S9. scRNA-seq analysis of the bronchoalveolar lavage fluid (BALF) of COVID-19. Related to Fig. 5.

The expression of genes identified in Figure 4 in the bronchoalveolar lavage fluid (BALF) COVID-19 patients or healthy donors. A total of 5 COVID-19 samples and 3 healthy control samples were used for RNA-seq analysis. The fold changes and adjusted P values of these genes between COVID-19 and healthy control donors were plotted.

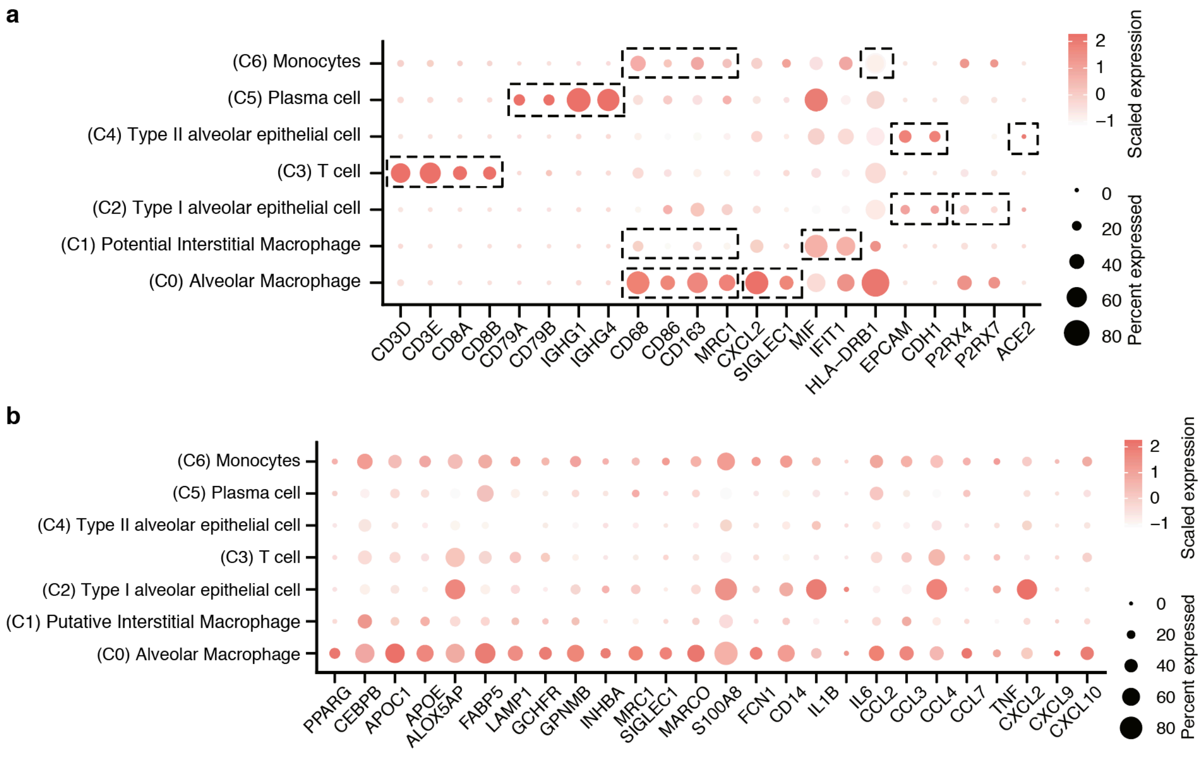

Supplementary Fig. S10. Dot plot shows the expression of cell-type-specific genes in each cluster of cells. Color represents relative expression. The size of the circle represents the percentage of expressed cells.

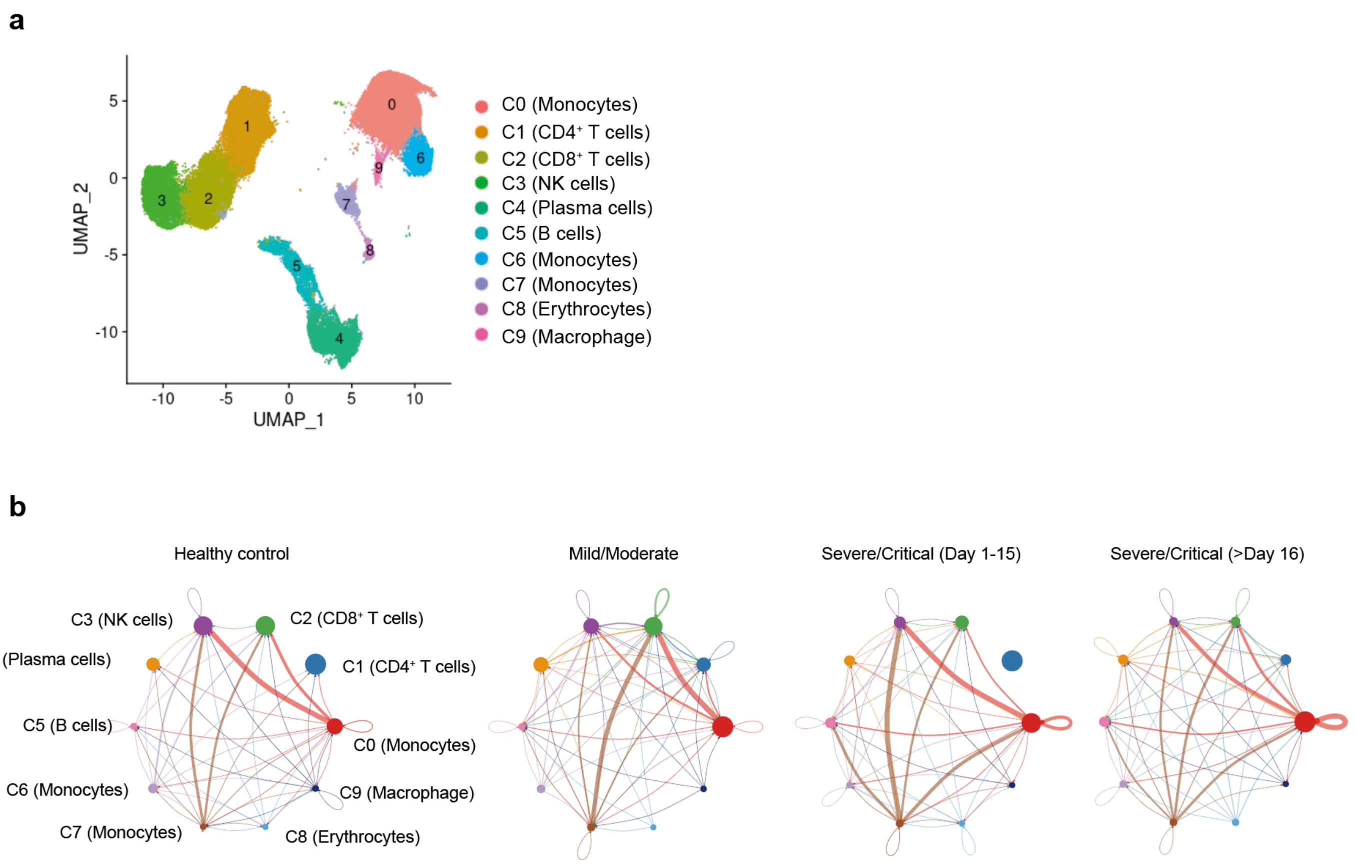

Supplementary Fig. S11. scRNA-seq analysis of PBMCs in COVID-19. Related to Fig. 6.

(**a**) Umap clustering of PBMC cells in COVID-19 and Healthy control donors. (**b**) Cell-cell interaction based signaling pathway networks in PBMC of healthy control donors, mild/moderate, severe/critical COVID-19 (Dpi 1-15 and Dpi 16-30). The CXCL signaling pathway networks were shown.

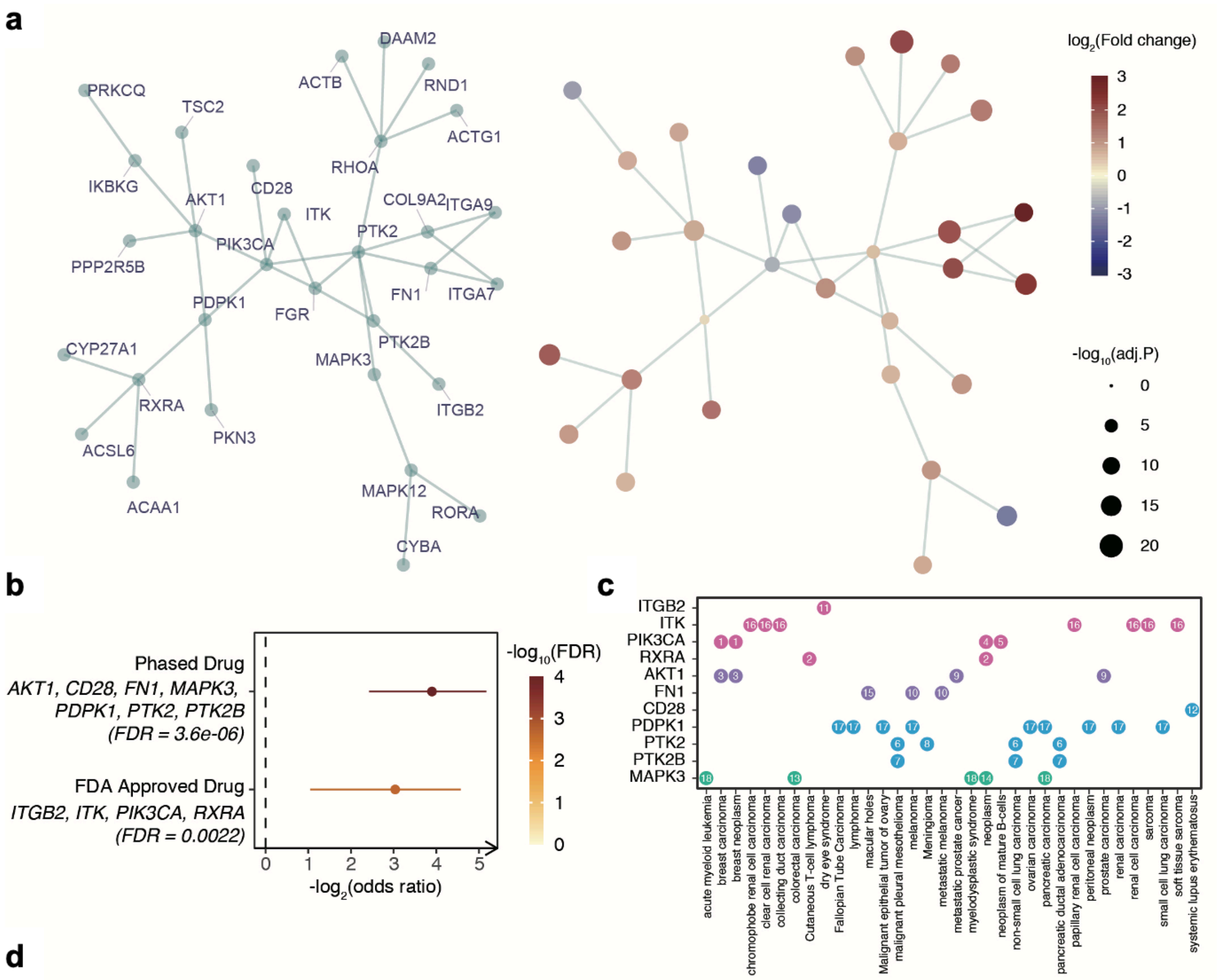

d

Index	Drug
1	*ALPELISIB (PI3-kinase p110-alpha subunit inhibitor)*
2	*BEXAROTENE (Retinoid X receptor agonist)*
3	*CAPIVASERTIB (Serine/threonine-protein kinase AKT inhibitor), IPATASERTIB (Serine/threonine-protein kinase AKT inhibitor)*
4	*COPANLISIB (PI3-kinase p110-alpha subunit inhibitor)*
5	*COPANLISIB HYDROCHLORIDE (PI3-kinase p110-alpha subunit inhibitor)*
6	*DEFACTINIB (Focal adhesion kinase 1 inhibitor)*
7	*DEFACTINIB (Protein tyrosine kinase 2 beta inhibitor)*
8	*GSK-2256098 (Focal adhesion kinase 1 inhibitor)*
9	*IPATASERTIB (Serine/threonine-protein kinase AKT inhibitor)*
10	*L19IL2 (Fibronectin binding agent)*
11	*LIFITEGRAST (Integrin alpha-L/beta-2 (LFA-1) antagonist)*
12	*LULIZUMAB PEGOL (T-cell-specific surface glycoprotein CD28 antagonist), TGN-1412 (T-cell-specific surface glycoprotein CD28 agonist)*
13	*MK-8353 (MAP kinase ERK1 inhibitor)*
14	*MK-8353 (MAP kinase ERK1 inhibitor), RAVOXERTINIB (MAP kinase ERK1 inhibitor), ULIXERTINIB (MAP kinase ERK1 inhibitor)*
15	*OCRIPLASMIN (Fibronectin proteolytic enzyme)*
16	*PAZOPANIB HYDROCHLORIDE (Tyrosine-protein kinase ITK/TSK inhibitor)*
17	*UCN-01 (3-phosphoinositide dependent protein kinase-1 inhibitor)*
18	*ULIXERTINIB (MAP kinase ERK1 inhibitor)*

Supplementary Fig. S12. Key networks related to SARS-CoV-2 infection and severity of COVID-19. Related to Fig. 6.

(**a**) Overview of the gene network identified through the integration of KEGG molecular interactions with gene expression changes in SC vs MM COVID-19. Gene nodes labelled by gene symbols (left), and coloured by expression changes, and sized by the expression significance (right). (**b**) Forest plot of approved or phased drug targets enriched in the gene network. The enrichment significance level was calculated according to Fisher's exact test (one-sided). FDA, US Food and Drug Administration. (**c-d**) Dot plot showing the network genes targeted by approved or phased drugs in disease indications (**c**). Dots are color-coded by clinical status, and the information on drugs and mechanisms of action is indexed and explained (**d**).
