# Supplementary material for: Integrating longitudinal clinical laboratory tests with targeted proteomic and transcriptomic analyses reveal the landscape of host responses in COVID-19
Source: Cell Discov. 2021 Jun 8;7:42. doi: 10.1038/s41421-021-00274-1 (PMC8185699; doi:10.1038/s41421-021-00274-1)
Supplement: Supplementary file 2 — Supplementary Table S1 [file 41421_2021_274_MOESM2_ESM.docx]

**Table S1. Demographic features of enrolled cases.**

|  | Mild/Moderate  (n=930) | Severe/Critical  (n=33) | P value ^a^ |
| --- | --- | --- | --- |
| **Age** | | | <0.0001 ^a^ |
| <=29 --no. (%) | 321 (34.5%) | 2 (6.1%) |  |
| 30-39 --no. (%) | 231 (24.8%) | 2 (6.1%) |  |
| 40-49 --no. (%) | 136 (14.6%) | 4 (12.1%) |  |
| 50-59 --no. (%) | 126 (13.5%) | 4 (12.1%) |  |
| 60-69 --no. (%) | 79 (8.5%) | 10 (30.3%) |  |
| >=70 --no. (%) | 27 (2.9%) | 11 (33.3%) |  |
| **Gender** | | | 0.02 ^a^ |
| Female --no. (%) | 380 (40.9%) | 7 (21.2%) |  |
| Male --no. (%) | 550 (59.1%) | 26 (78.8%) |  |
| **Viral test at initial diagnosis** | | |  |
| Positive --no. (%) | 930 (100%) | 33 (100%) |  |
| Negative --no. (%) | 0 | 0 |  |
| **Median length of hospital stay (IQR)** | 14 (10-19) ^b^ | 33 (23-53.5) ^b^ | <0.0001 ^b^ |
| **Anti-SARS-COV-2 S protein IgG (post 2-8 weeks of infection)** | | | 0.02 ^a^ |
| Strong --no./Total --no. (%) | 102/527 (19.4%) | 11/31 (35.5%) |  |
| Intermediate --no./Total --no. (%) | 294/527 (55.8%) | 18/31 (58.1%) |  |
| Low --no./Total --no. (%) | 131/527 (24.9%) | 2/31 (6.5%) |  |
| **Any Co-morbidities** | | |  |
| Hypertension --no. (%) | 89 (9.6%) | 13 (39.4%) | <0.0001 ^a^ |
| Diabetes --no. (%) | 40 (4.3%) | 9 (27.3%) | <0.0001 ^a^ |
| Coronary heart disease --no. (%) | 20 (2.2%) | 6 (18.2%) | <0.0001 ^a^ |
| Chronic hepatitis B --no. (%) | 10 (1.1%) | 1 (3.0%) | 0.30 ^a^ |
| Fatty liver disease --no. (%) | 12 (1.3%) | 1 (3.0%) | 0.39 ^a^ |
| Chronic renal disease --no. (%) | 3 (0.3%) | 1 (3.0%) | 0.02 ^a^ |
| **Acute Respiratory Distress Syndrome** | | | <0.0001 ^a^ |
| Yes --no. (%) | 0 (0.0%) | 26 (78.8%) |  |
| No --no. (%) | 930 (100.0%) | 7 (21.2%) |  |
| **Clinical outcome** |  |  | <0.0001 ^a^ |
| Discharged alive from hospital --no. (%) | 930 (100.0%) | 27 (81.8%) |  |
| Died --no. (%) | 0 (0.0%) | 6 (18.2%) |  |

^a^ Chi-square test; ^b^ two-sided Mann-Whitney U test; no.: Number of cases
