# Supplementary material for: Integrating longitudinal clinical laboratory tests with targeted proteomic and transcriptomic analyses reveal the landscape of host responses in COVID-19
Source: Cell Discov. 2021 Jun 8;7:42. doi: 10.1038/s41421-021-00274-1 (PMC8185699; doi:10.1038/s41421-021-00274-1)
Supplement: Supplementary file 4 — Supplementary Table S3 [file 41421_2021_274_MOESM4_ESM.docx]

| **Clinical Variables** | **Odds Ratio** | **95% Cl** | | **P value** |
| --- | --- | --- | --- | --- |
|  |  | **Lower** | **Upper** |  |
| **UNIVARIATE ANALYSIS** | | | | |
| Age | 1.095 | 1.066 | 1.124 | <0.0001 |
| Gender | 2.566 | 1.103 | 5.973 | 0.029 |
| Lymphocyte counts | 0.004 | 0.001 | 0.017 | <0.0001 |
| CD19^+^ cell counts | 0.985 | 0.979 | 0.99 | <0.0001 |
| CD3^+^ cell counts | 0.995 | 0.994 | 0.996 | <0.0001 |
| CD4^+^ cell counts | 0.992 | 0.99 | 0.995 | <0.0001 |
| CD8^+^ cell counts | 0.987 | 0.984 | 0.991 | <0.0001 |
| IL-6 | 1.043 | 1.025 | 1.062 | <0.0001 |
| IL-8 | 1.01 | 1.002 | 1.018 | 0.018 |
| IL-10 | 1.09 | 1.038 | 1.145 | 0.001 |
| **MULTIVARIATE ANALYSIS** | | | | |
| Lymphocyte counts | 0.025 | 0.005 | 0.124 | <0.0001 |
| IL-6 | 1.012 | 1.001 | 1.023 | 0.03 |
| Age | 1.035 | 0.999 | 1.073 | 0.06 |

**Table S3.** Statistical analyses of factors associated with disease severity.
